# Quantitative analysis of chromatin interaction changes upon a 4.3 Mb deletion at mouse 4E2

**DOI:** 10.1186/s12864-015-2137-5

**Published:** 2015-11-21

**Authors:** Cinthya J. Zepeda-Mendoza, Swagatam Mukhopadhyay, Emily S. Wong, Nathalie Harder, Erik Splinter, Elzo de Wit, Melanie A. Eckersley-Maslin, Thomas Ried, Roland Eils, Karl Rohr, Alea Mills, Wouter de Laat, Paul Flicek, Anirvan M. Sengupta, David L. Spector

**Affiliations:** Watson School of Biological Sciences, Cold Spring Harbor Laboratory, 1 Bungtown Road, Cold Spring Harbor, NY 11724 USA; Cold Spring Harbor Laboratory, 1 Bungtown Road, Cold Spring Harbor, NY 11724 USA; The European Molecular Biology Laboratory, European Bioinformatics Institute, Wellcome Trust Genome Campus, Hinxton, Cambridge, CB10 1SD UK; Department Bioinformatics and Functional Genomics, Biomedical Computer Vision Group, University of Heidelberg, BioQuant, IPMB, and German Cancer Research Center (DKFZ), Im Neuenheimer Feld 267, 69120 Heidelberg, Germany; Hubrecht Institute-KNAW & University Medical Center Utrecht, Uppsalalaan 8, 3584 CT Utrecht, The Netherlands; Epigenetics Programme, Babraham Institute, Babraham Research Campus, Cambridge, CB22 3AT UK; Center for Cancer Research, National Cancer Institute, National Institutes of Health, 50 South Drive, Bldg. 50, Rm. 1408, Bethesda, MD 20892 USA; Department of Physics and Astronomy, Rutgers, The State University of New Jersey, 136 Frelinghuysen Road, Piscataway, NJ 08854-8019 USA

## Abstract

**Background:**

Circular chromosome conformation capture (4C) has provided important insights into three dimensional (3D) genome organization and its critical impact on the regulation of gene expression. We developed a new quantitative framework based on polymer physics for the analysis of paired-end sequencing 4C (PE-4Cseq) data. We applied this strategy to the study of chromatin interaction changes upon a 4.3 Mb DNA deletion in mouse region 4E2.

**Results:**

A significant number of differentially interacting regions (DIRs) and chromatin compaction changes were detected in the deletion chromosome compared to a wild-type (WT) control. Selected DIRs were validated by 3D DNA FISH experiments, demonstrating the robustness of our pipeline. Interestingly, significant overlaps of DIRs with CTCF/Smc1 binding sites and differentially expressed genes were observed.

**Conclusions:**

Altogether, our PE-4Cseq analysis pipeline provides a comprehensive characterization of DNA deletion effects on chromatin structure and function.

**Electronic supplementary material:**

The online version of this article (doi:10.1186/s12864-015-2137-5) contains supplementary material, which is available to authorized users.

## Background

Chromatin organization in eukaryotic cells is associated with patterns of transcriptional activity and genomic stability (reviewed in [1–5]). Within the past decade, the development and diverse adaptations of the chromosome conformation capture (3C) technology [6] have unraveled the organization of chromatin in multiple cell types and organisms, advancing our understanding of chromosome structure at different length-scales (reviewed in [1]).

All 3C technologies use cross-linked chromatin to identify genomic interactions in the nuclear space, providing estimates of the frequency of contacts of specific regions with the rest of the genome (reviewed in [7]). Among the 3C-derived methodologies, the circular 3C (4C) technology was developed to target specific chromosomal regions (“viewpoints”) and identify their intra- and inter-chromosomal contacts (“captures”) [8]. Current 4C protocols use next-generation sequencing for the identification of captures (4C-seq) [9], and have been recently combined with paired-end sequencing (PE-4Cseq) to enable allele-specific identification of chromatin contacts through genotyping single nucleotide polymorphisms (SNPs) [10, 11]. Initial 4C analysis strategies identified significant viewpoint-capture interactions by running windowed statistical approaches to test whether the number of contacts was greater than expected against empirically computed background contact profiles [9, 11–13]. Other 4C analysis approaches have used power-law fits [14] or variance-stabilization with monotonous fits [15] to normalize the data and estimate background contact probabilities against which significant chromatin contacts are identified [9, 13–15].

However, chromatin fibers can be modeled as a polymer, i.e., “beads-on-a-string”, with nucleosomes as beads, and linker DNA as the string. Polymer entropy, or the random thermal exploration of all the spatial configurations of a polymer, can dictate the expectation of contact probabilities for a chromatin region. This probability of entropy-driven random contacts has been well-characterized in the polymer physics literature, and has been described as a power-law fall off of interactions between chromatin fragments with increasing genomic separation [16, 17]. This behavior has been clearly observed in 4C and Hi-C experiments [9, 12, 13, 18].

We developed a novel quantitative framework for the analysis of multi-viewpoint PE-4Cseq data firmly grounded on polymer physics. Our pipeline corrects for PE-4Cseq data biases, normalizes data, and computes the contact probability between a viewpoint fragment and all other fragments in the chromosome, allowing the quantitative testing of differences in chromatin contacts and chromatin compaction. In particular, this method is especially suited for the analysis of complex chromosome modifications such as copy number variants (CNVs). We applied this new approach to study chromatin interactions within and around a 4.3 Mb engineered deletion in mouse region 4E2 [19], which is orthologous to human 1p36 [19]. Allele-specific PE-4Cseq experiments of deletion and wild-type (WT) chromosomes revealed several local and long-range differentially interacting regions (DIRs) in the deletion-containing chromosome (*Df)*, as well as marked chromatin decompaction detected downstream of the deletion position. DIRs were enriched in differentially expressed (DE) genes as detected by RNA-Seq, as well as CTCF and Smc1 binding sites, proteins that had been previously characterized as structural determinants of chromatin organization [20–26].

Collectively, our work demonstrates the successful development and application of a quantitative PE-4Cseq analysis pipeline, and illustrates for the first time the complex and diverse effects of a DNA deletion on both the local and global signatures of chromatin organization and gene expression.

## Results

### General framework for the quantitative analysis of PE-4Cseq data

**Fig. 1:**
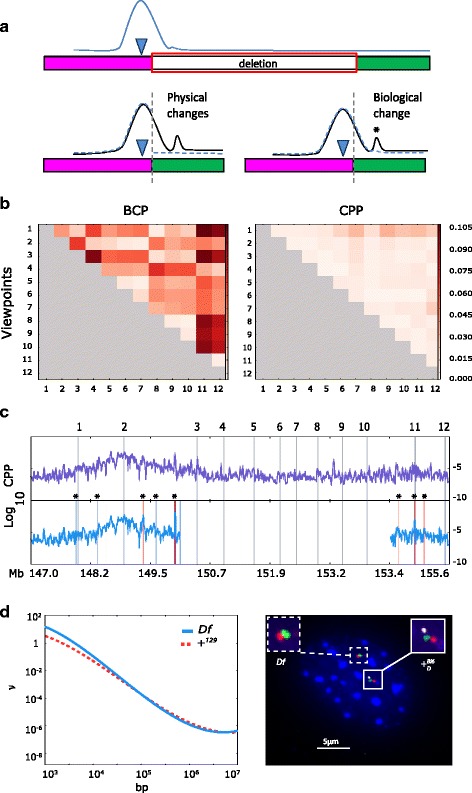
Study of chromatin contacts with polymer physics and key analysis phases. **a** *Top panel*: schematic representation of background chromatin interactions (blue line) for a viewpoint (blue inverted triangle) bordering a 4.3 Mb DNA deletion. Most of the interactions are expected to be surrounding the viewpoint given the smaller distance separating them. Notice how interactions further downstream of the viewpoint have a low contact probability. *Lower left panel*: upon deletion, two different genomic regions are joined together in physical proximity (pink and green rectangles), and the viewpoint presents a new interaction profile (black curve). Using current statistical methods, all the contacts established with the green region would be catalogued as differential, given their comparison to the original distal contact profile (dashed blue line). However, overlay of the previous WT background profile (blue dashed curve) shows that the majority of these contacts are simply following the expected WT background contact probability for the viewpoint (and are therefore physical changes in interaction), and only the peak marked with an asterisk would be considered as a genuine change in chromatin interactions (of a biological origin). **b** *Analysis Phase 1:* Result of bias-correction for a typical PE-4Cseq experiment on 12 viewpoints located on WT chromosome 4. Viewpoints index is on the *x* and *y* axis. The heatmap on the left is the relative asymmetry matrix $\frac{\left|{F}_{IJ}-{F}_{JI}\right|}{F_{IJ}+{F}_{JI}}$ in BCP per viewpoint where only the upper triangle is shown because the matrix is symmetric. The heatmap on the right is the relative asymmetry $\frac{\left|{P}_{IJ}-{P}_{JI}\right|}{P_{IJ}+{P}_{JI}}$ for *P*_*IJ*_ obtained after bias-correction. Notice the reduction in both row and column-wise biases and in the net asymmetry between viewpoints. Heatmaps are displayed in a log_10_ scale. **c** *Analysis Phase 2:* The CPP after bias correction for a typical viewpoint in a WT chromosome. *Analysis Phase 3:* A typical comparison between WT and deletion viewpoint CPPs to identify DIRs. The DIRs are shown in asterisks, and represented as vertical bands with widths proportional to their sizes. Color intensity is proportional to strength of signal, with reds for increase and blues for decrease of signal in the deletion versus WT comparisons. **d** *Analysis Phase 4.* Left panel: example spline-fit to the fall-off of CP (in log-log scale) against genomic viewpoint-fragment distances in a deletion (red) and WT (blue) datasets. The slope of the fit at 100 Kb is our local measure of compaction *ν*_*I*_, which may differ significantly in WT and deletion as illustrated in this panel for a typical viewpoint. Right panel: Example of a 3D DNA FISH experiment where a DIR pair was differentially-labeled using red and green probes to measure physical distances between them. The inclusion of a probe inside the deletion region (white), allows for the distinction of deletion (*Df*) and WT (+_*D*_^*Bl*6^) chromosomes within the same nuclei (blue)

When chromatin is modeled as a polymer, PE-4Cseq experiments provide the probability of each viewpoint fragment contacting any other fragment within the same chromosome; we call these frequencies the intra-chromosomal contact probability profile (CPP) of the viewpoint (Fig. 1a, top panel). Chromatin polymer analysis offers a major advantage compared to purely statistical approaches to identifying differential interactions upon the occurrence of DNA deletions; because in order to quantify the biological effects of a large-scale deletion on chromatin contacts, the expected changes in interaction of purely physical origin need to be distinguished from those of biological origin. Physical contributions include increased interactions between genomic regions newly proximal in the deletion chromosome— two proximal segments will interact more frequently compared to their WT counterparts owing to polymer entropy and not for any novel biological mechanisms (Fig. 1a, lower left panel). Biological contributions, on the other hand, may originate from changes in gene regulatory contacts, interactions mediated by chromatin architectural proteins, among others (Fig. 1a, lower right panel). Existing statistical-analysis methods are not designed for such discrimination because the null expectation of signal is set by sequencing technology considerations alone and not by a polymer model which incorporates the physical effects systematically.

We designed a pipeline optimized for bias-correction, data normalization, and quantitative comparisons in multi-viewpoint PE-4Cseq data. The pipeline provides an optimal estimate of CPP from the biased and noisy measurements in PE-4CSeq experiments. These CPPs include both background non-specific interactions between a viewpoint and any other region (arising from polymer entropy), and the specific interactions that have a biological origin. CPP signal differences are key in drawing meaningful conclusions on distinct control and test experimental conditions.

We set the null expectation, of entirely entropic origin, for each viewpoint’s CPP using a generalized Gaussian “beads-on-a-string” polymer model of chromatin (see Methods for model details). In this model, 4C viewpoints which are neighbors along the chromatin fiber (*I*, *I* + 1) are connected by Gaussian springs with spring constants *k*_*I*,*I* + 1_. These spring constants are a measure of the strength of tethering of one chromatin fragment to another mediated by the intervening chromatin fiber, and exhibit a power-law scaling for intermediate genomic separations (100 Kb-10 Mb). The CPP, represented by *P*_*IJ*_, has the scaling form ${P}_{IJ}\sim {s}_{IJ}^{\nu_I},$ where *s*_*IJ*_ is the genomic separation between viewpoint positions *I* and *J*, and *ν*_*I*_ is the local scaling exponent. The CPP typically falls off with increasing genomic separation of interacting fragments—entropic interactions between distal fragments are rare—therefore *ν*_*I*_ is always negative. Different polymer models of chromatin have different scaling. For example, random-walk (Gaussian) chromatin polymers exhibit a uniform scaling exponent $\left(\nu = -\frac{3}{2}\right),$ while Hi-C data analysis has shown that most eukaryotic chromatin is on average more compact (*ν* = − 1) leading to the hypothesis of a non-equilibrium (“fractal globule”) model of chromatin [16, 18]. We used *ν*_*I*_ as a measure of viewpoint-specific chromatin compaction and its variability upon deletion occurrence, as presented below.

This new data analysis pipeline comprises the following key phases:

#### Phase 1: Bias correction

The ideal output from any PE-4Cseq measurement is the CPP of the chromatin polymer. However, several biases corrupt the straightforward measurement of the interaction profile. Numerous biases have been reported in previous Hi-C and 4Cseq data analyses, and these include genomic restriction fragment lengths, fragment GC content, and amplification primer efficiencies [12, 13, 27]. The second class of bias is specific to multi-viewpoint 4C experiments and originates from variability in viewpoint sample preparation and sequencing amplification steps.

We removed biases without modeling them individually by their technical or biological sources. We first normalized the capture data for each experiment by the product of viewpoint and fragment lengths, and call this the biased contact probability (BCP) (Fig. 1b). This normalization corrects for the bias that a longer polymer fragment has more contacts directly proportional to its length. We modeled the BCP between two viewpoints as *F*_*IJ*_ = *C*_*I*_*C*_*J*_*K*_*I*_*P*_*IJ*_, where *C*_*I*_ and *C*_*J*_ are the overall bias factors corresponding to the (*I*, *J*) viewpoint sequences, *K*_*I*_ is the overall bias factor for the capture experiment of viewpoint *I*, and *P*_*IJ*_ is the average CPP for viewpoints *I* and *J*. Similarly, for the experiment corresponding to viewpoint *J*, the normalized capture data is *F*_*JI*_ = *C*_*I*_*C*_*J*_*K*_*J*_*P*_*IJ*_; from these equations, note that only the experiment bias factor *K*_*J*_ is distinct. We then solved the linear set of equations (in logarithm space) to compute the bias factors *C* and *K* from *F*_*IJ*_ and *F*_*JI*_. The unbiased estimate of the contact probability, *P*_*IJ*_, should be symmetric for both *I* and *J* viewpoints. We performed this bias correction using only nearest and next-nearest-neighbor viewpoint pairs, and observed that biases for all other viewpoint pairs are significantly reduced and *P*_*IJ*_ is very close to being symmetric, whereas *F*_*IJ*_ is clearly not (Fig. 1b and Additional file 1: Figure S1), attesting to the consistency of bias correction. Given that the bias modeling introduces 2*n* parameters for *n* viewpoints, there are at most *n*(*n* − 1) observable *F*_*IJ*_. Therefore, at least four viewpoints to perform bias correction analyses are needed.

#### Phase 2: Computation of a smoothed CPP

We used the bias factors to estimate the unbiased CPP per viewpoint and for all their contacting intra-chromosomal fragments (captures) (Fig. 1c). The CPP for viewpoint *I* and fragment *α* is defined as ${P}_{I\alpha }=\frac{F_{I\alpha }}{C_I{K}_I},$ where *F*_*Iα*_ is the BCP, *C*_*I*_ is the overall viewpoint *I* bias factor, and *K*_*I*_ is the bias factor for the PE-4Cseq experiment of viewpoint *I*. Once the raw CPP per viewpoint was obtained, we thresholded and smoothed it using a Gaussian kernel of standard-deviation 20 Kb (total filter size 80 Kb) for whole chromosome contact comparisons. The 20 Kb standard deviation is in the upper range of viewpoint sizes (~0.5-20Kb) in our experiments, and is the genomic resolution of the PE-4Cseq measurements.

#### Phase 3: Discovering Differentially Interacting Regions (DIRs)

The smoothed CPP per viewpoint was used to compute the differential relative contact probability (DRCP) signals, defined as (log *P*_*del*_ − log *P*_*WT*_)/log *P*_*WT*_, between *Df* and + _*D*_^*Bl*6^ (*del*) chromosomes relative to the WT CPPs for +^*129*^ and + ^*Bl6*^ in two biological replicates (Fig. 1c). In order to reduce Poisson noise in mapped read-counts we only considered captured fragment read counts greater than a threshold (5 reads per restriction fragment). We added a pseudo-count of 1 to all viewpoint-fragment pairs so that missing data does not lead to false DRCP signals. We analyzed the statistical significance of all DRCP signals at a level of > =5 % interaction probability differences as follows: We first considered a window bracketing the location of each DRCP, with a window size equal to the size of the Gaussian smoothing filter (80 kb). The raw CPP data from *del* and *WT* viewpoints were subjected to the Mann–Whitney *U* test at each window, to check whether their difference was statistically significant (*p*-value < 0.05). We performed this testing independently for the two biological replicates, and reported DRCPs only when they were present (within the window size) in *both* replicates. The regions of the chromosome with significant DRCP signals were catalogued as differentially interacting regions (DIRs).

#### Phase 4. Measurement of local chromatin compaction

We characterized local chromatin compaction for the chromosomes in *Df/*+ _*D*_^*Bl*6^ and +^*129*^/+^*Bl6*^ by fitting a smoothing spline to the logarithm of contact probability against the logarithm of genomic separation. The slope of this curve at 100 Kb defines our local scaling exponent *ν*_*I*_ for viewpoint *I* and is a local measure of chromatin compaction. The length scale of 100 Kb is chosen by observation of the data-rich linear region of the CPP fall-off (Fig. 1d, left panel). Within this range, topologically associating domains (TADs) have been discovered [21, 28], and our analysis reveals possible changes in higher-order chromatin organization upon the occurrence of a deletion or any other CNV, testable by additional experiments such as 3D DNA FISH (Fig. 1d, right panel).

The development of a pipeline grounded in polymer physics for the analysis of multi-viewpoint PE-4Cseq data is an important contribution to those surveying chromatin conformation under diverse experimental conditions, including CNVs. In the next sections, we apply this pipeline to the investigation of chromatin organization upon a 4.3 Mb deletion in mouse 4E2, and offer an integrative picture of the effects of a large-scale chromatin deletion on chromosome conformation and transcriptional output.

### Allele-specific regions of differential contact probabilities in *Df/* + _***D***_^***Bl***6^ MEFs

**Fig. 2:**
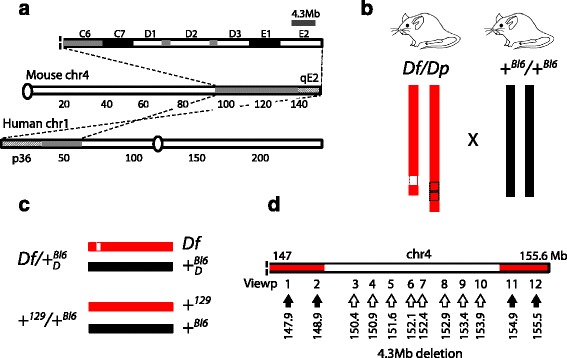
4E2 and 1p36 orthologous regions, and engineered mouse models used. **a** Human 1p36-p31.3 and mouse 4C5-E2 bands are orthologous (dashed lines). 1p36 and 4E2 are shown in dashed rectangles in their respective orthologous chromosome segments (grey rectangles). The 4.3 Mb deletion region inside mouse E2 is the focus of this study (top grey rectangle). **b** First generation (F1) *Df/Dp* male chimeras engineered in 129S5/SvEv^Brd^ ES cells were mated with WT C57BL/6 J females to obtain heterozygote F1 *Df/*+ _*D*_^*Bl*6^ progeny. **c** *Df/*+ _*D*_^*Bl*6^ and +^*129*^/+^*Bl6*^ genotypes with corresponding chromosome name conventions used in this study. *Df* corresponds to the engineered 129S5/SvEv^Brd^ chromosome 4 harboring the deletion in *Df/*+ _*D*_^*Bl*6^ MEFs, while + _*D*_^*Bl*6^ corresponds to the WT C57BL/6 J chromosome 4. In *+* ^*129*^ */+* ^*Bl6*^ MEFs, +^*129*^ corresponds to the 129S5/SvEv^Brd^ WT chromosome 4 and *+* ^*Bl6*^ corresponds to the WT C57BL/6 J chromosome 4. **d** The PE-4Cseq viewpoint positions along chromosome 4 selected for this study are indicated by arrows. Two viewpoints span ~2.5 Mb upstream of the deletion start, eight are located inside the deletion, and two cover ~1 Mb downstream of the deletion end

4E2 is a gene-rich region in mouse orthologous to human 1p36 (Fig. 2a). 1p36 is an interesting genomic region where heterozygous deletions of different sizes are often present in a wide variety of cancers [29] and can originate a mental retardation syndrome known as Monosomy 1p36 [30]. Given the importance of 1p36 deletion CNVs in humans, we decided to study a chromosome-engineered mouse model harboring a 4.3 Mb deletion in 4E2 [19].

We investigated chromatin organization in the heterozygous F1 genotype *Df/*+ _*D*_^*Bl*6^. *Df* is the chromosome 4 from the 129S5/SvEv^Brd^ mouse strain with an engineered 4.3 Mb deletion in 4E2 [19] (Fig. 2b). + _*D*_^*Bl*6^ denotes a WT C57BL6/J chromosome 4 copy. Chromosomes derived from the 129S5/SvEv^Brd^ and C57BL6/J mouse strains can be easily distinguished through genotyping single nucleotide polymorphisms (SNPs) [31], therefore, allele-specific analyses of 4E2 chromatin contacts are feasible by PE-4Cseq in *Df/* + _*D*_^*Bl*6^ and their corresponding +^*129*^/+^*Bl6*^ WT controls (Fig. 2c and Additional file 1: Figure S2). The study of heterozygote genotypes allowed us to investigate the architectural consequences of the deletion chromosome in direct comparison to its WT counterpart.

Mouse embryonic fibroblasts (MEFs) were derived from F1 *Df/*+ _*D*_^*Bl*6^ mice, as well as +^*129*^/+^*Bl6*^ controls. A total of 12 viewpoints spanning 4E2 were selected (Fig. 2d and Additional file 2: Table S1A), and the presence of reported SNPs [31] validated by Sanger sequencing (Additional file 2: Table S1B). All viewpoints were amplified from two biological replicates of *Df/*+ _*D*_^*Bl*6^ and +^*129*^/+^*Bl6*^ MEFs, and reads trimmed and mapped against a reduced *HindIII* restriction fragment database (Additional file 2: Table S1C, D). We concentrated our analysis on viewpoints located outside of the deleted region (1, 2, 11, 12) (Fig. 2d), as they allowed us to directly interrogate the consequences of a DNA deletion on local and long-range chromatin interactions. However, DIR analysis of viewpoints within the deletion region in WT chromosomes was also performed, and both *Df* and WT DIR data are available through GEO Series accession number GSE64360 (see Methods).

**Fig. 3:**
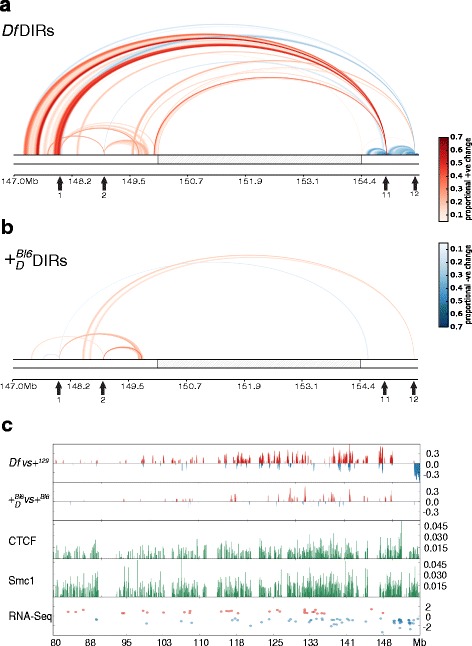
*Df* and + _*D*_^*Bl*6^ DIRs along chromosome 4. **a** “Rainbow plot” for the 4E2 region representing the DIRs for the comparison between *Df* and +^*129*^, and between + _*D*_^*Bl*6^ and + ^*Bl6*^ computed with a 20 Kb smoothing factor for the viewpoints surrounding the deletion **b**. Rainbow plots were used to visualize both short and long-range interaction changes. The arcs represent increased interaction (red) or decreased interaction probabilities (blue), with the strength of signal color coded. For visual clarity we only show signals with strength >10 %, and do not include contacts inside the deletion region for + _*D*_^*Bl*6^ in **b**. Grey arrows annotate positions for viewpoints 1, 2 (upstream of deletion) and 11, 12 (downstream of deletion, towards telomeric end). **c** Positions of DIRs for all viewpoints 1, 2, 11 and 12 are compared against structural protein binding sites. First track is for *Df* and +^*129*^ comparisons and second track is for + _*D*_^*Bl*6^ and + ^*Bl6*^ comparison. DIRs with increased/decreased contact probabilities compared to WT are plotted in red/blue. Shown in the third and fourth tracks (green bars) are the running average over 20 Kb window for CTCF and Smc1 binding site footprint, respectively. The fifth track displays fold change for *Df/*+ _*D*_^*Bl*6^ DE genes, color coded (red, over-expressed in Df/+ _*D*_^*Bl*6^ MEFs; blue, over-expressed in +^129^/+^Bl6^ MEFs)

There are 84 DIRs detected for viewpoints 1, 2, 11, and 12 in *Df* when compared against +^*129*^ (Fig. 3a, c and Additional file 1: Figure S3 top panel). These DIRs add up to ~34 Mb (~22 %) of chromosome 4 sequence, have a minimum size of 160 Kb, a median size of 260 Kb, and a maximal size of 1.495 Mb. The changes observed for viewpoints 1, 2, and 11 mostly involve increases in interaction with surrounding sequences; viewpoint 12 displays a decrease in contact probabilities for their identified DIRs (Fig. 3a and Additional file 3: Table S2). As can be seen in the top panel of Additional file 1: Figure S3, *Df* DIRs are scattered along chromosome 4, with the majority of the DIRs neighboring the deletion.

We also performed the reciprocal + _*D*_^*Bl*6^ and + ^*Bl6*^ WT comparison in order to assess the level of uniqueness of *Df* DIRs, and to test whether the 4.3 Mb deletion in *Df* had a *trans* influence on the chromatin contacts of its WT counterpart. Analysis of viewpoints 1, 2, 11, and 12 revealed a total of 63 DIRs between + _*D*_^*Bl*6^ and + ^*Bl6*^ chromosomes (Fig. 3b, c and Additional file 1: Figure S3 s panel), and their sequences add up to ~15 Mb (~9.6 % of chromosome 4). + _*D*_^*Bl*6^ DIRs tend to be smaller compared to *Df* DIRs, and have a minimum size of 160 Kb, a median size of 213 Kb, and a maximal size of 626 Kb. The changes observed for these viewpoints mostly involve increases in contact probabilities (Fig. 3b, c and Additional file 4: Table S3). Similar to *Df* DIRs, + _*D*_^*Bl*6^ DIRs are scattered along chromosome 4, with the highest number present surrounding the deletion (Additional file 1: Figure S3, second panel). There are 55 *Df* DIRs that intersect with + _*D*_^*Bl*6^ DIRs, covering ~9 Mb of chromosome 4 sequence (~6 %) (Additional file 5: Table S4A). The high overlap ratios between *Df* and + _*D*_^*Bl*6^ DIRs suggests the existence of *trans* mechanisms of chromatin architecture regulation common to both chromosome 4 homologues which are affected after the occurrence of the 4.3 Mb 4E2 deletion. In order to query the DIRs segments which are unique to *Df*, we eliminated the genomic regions where the *Df* and + _*D*_^*Bl*6^ DIRs overlap. There are 42 unique *Df* DIR segments covering ~12 Mb (~7.7 % chromosome 4) with a median size of 225.5 Kb and maximal size of 1.195 Mb (Additional file 5: Table S4B). Similarly, there exist 13 DIR segments that are unique to + _*D*_^*Bl*6^, covering ~3 Mb of sequence (~2 % chromosome 4) with a median size of 195 Kb and a maximal size of 454 Kb (Additional file 5: Table S4C).

A number of 3C and Hi-C studies have implicated various proteins as structural determinants of the 3D organization of the mammalian genome. CTCF and cohesin have been shown to be boundary proteins between TADs, while Mediator complex plays important roles in loop formation for correct gene activation [20–26]. We used previously published CTCF, Med1, Med12, and Smc1 MEF binding datasets [20] to query the overlap between structural protein binding sites and DIR positions. We first estimated the density of structural protein binding sites at the resolution matching the DIR signal resolution (20 Kb). Reassuringly, we observed that several DIRs align with regions dense in binding sites in a whole chromosome view (Fig 3c). Using random sampling of all structural protein binding sequences along chromosome 4, we observed that *Df* and + _*D*_^*Bl*6^ DIRs are enriched in CTCF and Smc1 binding sites (*p*-val < 0.001) (Additional file 6: Table S5A-F). These observations suggest that alterations observed in the contact probability of DIRs could be mediated by CTCF and Smc1, and their binding may be affected by differential expression or by other chromatin remodeling genes in *Df/*+ _*D*_^*Bl*6^ MEFs (see discussion).

Altogether, the presented DIR data indicate the large amount of chromatin contact variation arising after the occurrence of a 4.3 Mb DNA deletion in mouse 4E2. Up to 22 % of chromosome 4 sequence is catalogued as DIRs with > 5 % changes in contact probability between *Df* and +^*129*^ chromosomes; interestingly, this extensive network of chromatin contact changes was identified by analyzing the intra-chromosomal contacts of only 4 viewpoints surrounding the deletion. The identification of *Df*-shared + _*D*_^*Bl*6^ DIRs suggests that *trans* alterations in chromatin interactions occur in *Df/*+ _*D*_^*Bl*6^ MEFs, which extend along the chromosome 4 sequence and could be mediated by CTCF and Smc1 protein binding or additional chromatin remodelers (see Discussion).

### Changes in local chromatin compaction in the *Df* chromosome

Each viewpoint’s contact probability falls off with increasing genomic separation, ${P}_{IJ}\sim {s}_{IJ}^{\nu_I},$ where *s*_*IJ*_ is the genomic separation between viewpoint positions *I* and *J*, and *ν*_*I*_ is the local scaling exponent. More compact chromatin regions have higher *ν*_*I*_ values, while less compact zones have smaller *ν*_*I*_’s. In addition to the identification of chromatin contact differences computed at the 20 Kb scale, our pipeline characterizes *ν*_*I*_, a signature of higher-order chromatin organization at intermediate length scales (100 Kb to 1 Mb).

**Fig. 4:**
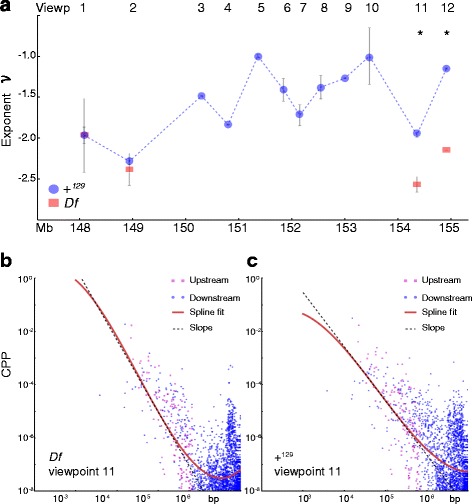
Computed *ν* per viewpoint. **a** *ν* values were computed for each viewpoint in Df (red rectangles) vs. the average of + ^Bl6^, +^129^ and + _*D*_^*Bl*6^ (blue circles) chromosomes, with error bars determined from the two biological replicates. Values of *ν* < − 3/2 corresponds to less compact state than expected from Gaussian behavior, whereas *ν* > − 3/2 corresponds to more compact states. Notice the decrease in overall compaction for Df and most significantly at the telomeric end. **b** Contact probability fall-off for viewpoint 11 is shown for Df and (**c**) WT values

We asked whether the scaling exponent ν_I_varies for each viewpoint *I* in *Df/*+ _*D*_^*Bl*6^ MEFs. Overall, considerable variation was observed between the scaling exponents for the 12 viewpoints along the + _*D*_^*Bl*6^ chromosome, in agreement with observed qualitative variation in local compaction in Hi-C data heatmaps [18, 28]. Significant differences in *ν*_*I*_ values are observed for viewpoints 11 and 12 in the *Df* chromosome, which are less compact than expected upon deletion (Fig. 4a, b, c). Interestingly, comparison of *ν*_*I*_ values between + _*D*_^*Bl*6^ and + ^*Bl6*^ viewpoints also revealed compaction differences for these viewpoints, however their differences are not as dramatic as seen in *Df* (Additional file 1: Figure S4). Notably, 3 viewpoints inside the deletion region in + _*D*_^*Bl*6^ also displayed significant differences in their *ν*_*I*_ values. These observations suggest that the *trans* effects of the deletion in chromatin contact differences are also reflected on higher-order chromosome structure in both *Df* and + _*D*_^*Bl*6^ chromosomes, and these may be caused by either changes in gene expression or by physical factors affecting chromosome organization in *Df/*+ _*D*_^*Bl*6^ MEFs.

Viewpoints 11 and 12 reside near the telomere of chromosome 4. Given the significant decrease in compaction for these viewpoints in *Df* compared to + _*D*_^*Bl*6^, a possible explanation is that the observed *Df* decompaction is a direct consequence of the physical deletion of a chromosome fragment. We hypothesized that tethering points bordering the deletion region may cause the telomeric end and adjacent upstream sequences to remain in their original preferred positions, subsequently stretching the intervening sequence after deletion. Such tethering points could be lamina-associated domains (LADs), important features of nuclear architecture and genomic regulation [32, 33]. Analysis of published LAD positions identified in 3 T3 MEFs [34] revealed the presence of only LAD-free regions bordering the deletion (147–150 Mb and 154.4–155.6 Mb on chromosome 4) (Additional file 7: Table S6). However, 10 LADs exist within a 1.3 Mb segment inside the deletion sequence (~25 % of the deletion size). Intriguingly, it is within this segment where the 3 viewpoints inside the deletion region in + _*D*_^*Bl*6^ also displayed significant differences in chromatin compaction (Additional file 1: Figure S4 and Additional file 7: Table S6). It is therefore possible that the deletion of tethering points within the deletion region in *Df* chromosomes allows for chromatin to be less compact.

### Validation of changes in chromatin interactions by 3D DNA FISH

After the identification of DIRs and the observed chromatin decompaction in *Df* chromosomes using our pipeline, we sought to validate these results using 3D DNA fluorescence *in situ* hybridization (FISH). 3D DNA FISH experiments provide direct measurements of physical distances separating any pair of genomic loci inside the nucleus, which can be used to estimate their contact frequencies (see Methods). This allows the assessment of whether DIRs were actually present in *Df* and WT chromosomes, and the quantitation of interaction changes at the single cell level in comparison to PE-4Cseq results.

**Fig. 5:**
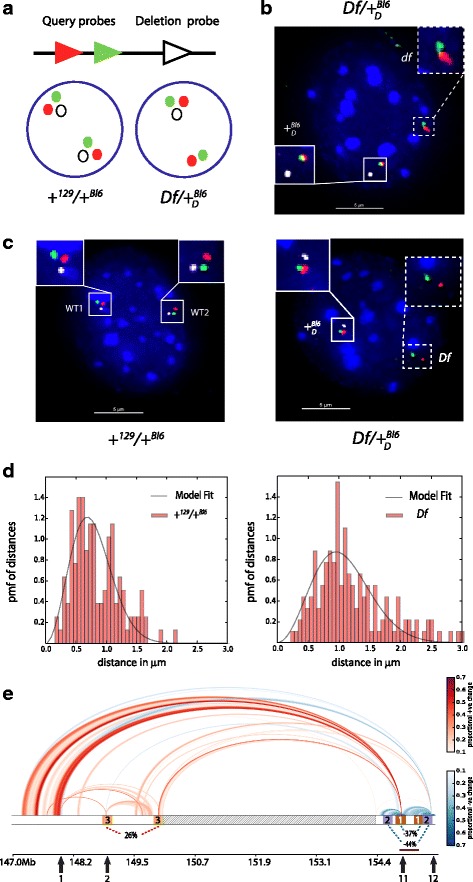
3D DNA FISH validations of PE-4Cseq data. **a** 3D DNA FISH strategy used. Green and red triangles represent query probes along the chromatin fiber separated at fixed distances. The white triangle represents the deletion probe. Red/green probes can also be located bordering the deletion probe. The Df chromosome is distinguished by the absence of the deletion probe, as shown in the nuclear representations of Df/+ _*D*_^*Bl*6^ and +^129^/+^Bl6^ genotypes. **b** Projection of a 3D DNA FISH image for control BACset4 using Df/+ _*D*_^*Bl*6^ MEFs. Red and green probes are the query probes, while white probe is the deletion probe. Hybridization images are zoomed inside white rectangles. Note the absence of the deletion probe for one of the chromosomes, classified as Df with our algorithm (dashed rectangle). **c** Representative 3D DNA FISH image projections for +^129^/+^Bl6^ and Df/+ _*D*_^*Bl*6^ nuclei for BACset2 probes. Probe colors are as described in **b**. Df chromosome probes are shown inside the white dashed rectangle, and WT probes shown in rectangle. **d** Query probe distance distributions for BACset2 (brown underline in Fig. 5e) for both +^129^/+^Bl6^ chromosomes (left panel) and Df (right panel). The black line indicates the model fit to the probability mass function (PMF). Notice the increase in typical spatial separations as well as a broader distribution in Df, in agreement with the observed decrease in contact probabilities in PE-4Cseq. **e** Rainbow plot of the zoomed in region near the deletion in Df chromosome. Enrichment and depletion of long-range interactions are color coded as in Fig. 3. The black arrows represent viewpoint positions; dashed box of the chromosome bar is the deletion region; the three distinct colored boxes are the three query probe pairs, also identified by numbers; the dashed rectangle inside the deletion region is the deletion probe. The dashed triangles represent query probe interaction changes as detected from FISH experiments, with enrichment in red and depletion in blue with associated proportional changes in interaction reported as percentages. Notice the agreement between enrichment (red) and depletion (blue) between PE-4Cseq and DNA FISH interactions

3D DNA FISH experiments were performed using two different fluorescently labeled bacterial artificial chromosome (BAC) probes overlapping DIR positions (‘query probes’), with a third BAC probe inside the deletion region (‘deletion probe’) (Fig. 5a, b). The deletion probe was used to distinguish the *Df* chromosome, and its location is identical for all experiments. Four representative *Df* regions were selected for analysis by 3D DNA FISH: three of these regions were classified as DIRs, some of which exhibited chromatin decompaction (BAC sets 1–3), while the fourth region (BAC set 4) tested a specific interacting pair observed in our PE-4Cseq data not classified as a DIR but enriched in CTCF and Smc1 binding sites (Fig. 5e and Additional file 8: Table S7A, B, C). Over a hundred nuclei were analyzed per 3D DNA FISH experiment, and query probe distances were measured in *Df/*+ _*D*_^*Bl*6^ and +^*129*^/+^*Bl6*^ MEFs.

Our analysis identified a qualitative agreement between the contact changes in DIRs derived from PE-Cseq and the changes in spring constant for 3D DNA FISH experiments (Fig. 5e). Query probes from FISH experiments testing DIRs with a decrease in contact probability and chromatin decompaction (BAC set 1, 2) exhibited larger separation distances compared to +^*129*^ measurements (Fig. 5c, d, e). Similarly, query probes with a reported PE-4Cseq increase in contact probabilities (BAC set 3) displayed smaller 3D FISH physical separation distances compared to +^*129*^ measurements (Additional file 1: Figure S5). Our control FISH experiment (BAC set 4) displayed a much narrower and almost identical distribution of distances between query probes in both WT (+^*129*^/+^*Bl6*^) and deletion (*Df/*+ _*D*_^*Bl*6^) chromosomes compared to the expectation for similar genomic separation (Fig. 5b and Additional file 1: Figure S5). This is indicative of pronounced long-range interactions between these two regions, in agreement with the peak in contact probability observed in our PE-4Cseq data. Given the enrichment of CTCF and Smc1 protein binding sites at these regions, it is possible that such stable contacts may be mediated by these proteins. Taken together, 3D DNA FISH results validate our PE-4Cseq analysis framework, and point to *bona fide* changes in contact probabilities arising after the occurrence of a 4.3 Mb deletion in mouse chromosome 4.

### Impact of chromatin conformation changes on 4E2 gene expression

CNVs are known to affect expression patterns of distal and neighboring genes [35–37]. Some of these effects may arise through changes in chromosome conformation: at the chromatin level, CNVs can potentially disrupt associations of gene promoters and their regulatory elements, affect topologically associating domain (TAD) boundaries [21, 28], and/or join differentially regulated regions. To assess the potential impact of DIRs on *Df/*+ _*D*_^*Bl*6^ gene expression, we analyzed the transcriptome of *Df/*+ _*D*_^*Bl*6^ MEFs through RNA sequencing (RNA-seq) [38–40].

**Fig. 6:**
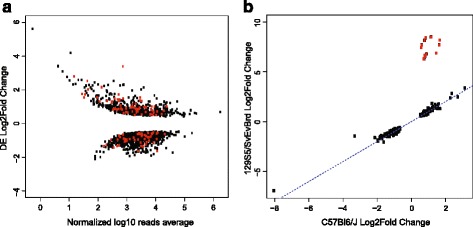
**a** log2fold ratios (y axis) and their corresponding average of normalized counts (x axis) for all DE genes between Df/+^Bl6^ and +^129^/+^Bl6^ MEFs. Positive values indicate higher expression in +^129^/+^Bl6^ MEFs, while negative values indicate higher expression in Df/+^Bl6^ MEFs. Shown in red are all the DE genes present on chromosome 4. **b** log2fold ratios for DE 129S5/SvEv^Brd^ alleles (y axis) and DE C57Bl6/J alleles (x axis) in Df/+^Bl6^ MEFs. Notice the high degree of correlation between the allelic log2fold changes when compared to a perfect correlation score (1, blue dashed line). Genes shown in red fall within the deletion sequence, and therefore display a different behavior compared to other alleles

*Df/*+ _*D*_^*Bl*6^ and +^*129*^/+^*Bl6*^ MEFs RNA-seq experiments revealed 1345 differentially expressed (DE) genes between both genotypes (Fig. 6a and Additional file 9: Table S8). 118 of these are chromosome 4 genes, a higher number than expected by chance (Fisher’s exact test, *p* = 0.017). Enrichment analysis revealed 4E2 and 4E as the cytogenetic bands with the most significant DE clustering locations in *Df/*+ _*D*_^*Bl*6^ MEFs (*p* = 1.48e–11, *p* = 3.26e–08 respectively). Interestingly, the 4D region, directly upstream of 4E, was the next most significant DE clustering location in the genome (*p* = 2.27e–08). 34 % of DE genes in chromosome 4 are contained within *Df* DIRs, and 24 % of DE genes are contained within + _*D*_^*Bl*6^ DIRs (Fig. 3c). Both *Df* and + _*D*_^*Bl*6^ DIRs and DE combined genes overlap ratios are significant (*p* < 0.05) (Additional file 6: Table S5B,D). Very interestingly, CTCF gene expression is increased in *Df/*+ _*D*_^*Bl*6^MEFs (0.5 log2fold change). Similarly, Gene Ontology (GO) analysis into cellular function for *Df/*+ _*D*_^*Bl*6^MEFs revealed 26 genes associated with “condensed nuclear chromosome” (Additional file 10: Table S9).

Given our use of a 129S5/SvEv^Brd^ and C57BL6/J heterozygote genetic background, we were able to perform allele-specific expression analysis in *Df/*+ _*D*_^*Bl*6^ MEFs. We detected 257 DE 129S5/SvEv^Brd^ alleles, 39 of them in chromosome 4 (Fig. 3c and Additional file 11: Table S10). In addition, 326 DE C57BL6/J alleles were detected, with 39 located in chromosome 4 (Fig. 3c and Additional file 12: Table S11). 27 DE alleles are shared between the 129S5/SvEv^Brd^ and C57BL6/J alleles, 12 of them inside the deletion position. Both allelic DE sets cluster in the 4E2 and 4E region (*p* < 0.001), where the deletion resides. Interestingly, despite the elimination of only the 129S5/SvEv^Brd^ allele inside the deletion region, 198 DE genes located in diverse genomic regions along all mouse chromosomes were mis-regulated at both alleles, and their allelic fold changes were highly correlated (*ρ* = 0.95, *p* = 2.2e–16), with the exception of the deleted genes located inside the deletion region, which exhibit a higher fold change for the 129S5/SvEv^Brd^ allele only (Fig. 6b). This observation is indicative of *trans* effects in transcription, where mRNA levels are regulated similarly between alleles. This phenomenon is reminiscent of the *trans* effects on chromatin contacts observed for the + _*D*_^*Bl*6^ chromosome in *Df/*+ _*D*_^*Bl*6^ MEFs. In fact, 18 % of the C57Bl6/J DE alleles on chromosome 4 are contained within + _*D*_^*Bl*6^ DIRs (Additional file 6: Table S5C). Whether *trans* effects in gene expression induce the appearance of DIRs in the + _*D*_^*Bl*6^ chromosome, or vice versa, remains to be tested in future experiments (see Discussion).

## Discussion

Previous 4C-Seq analysis methodologies have been based on various statistical models [9, 11–15]. Here, we have developed a pipeline based on polymer physics specifically targeted for analyzing 4C sequencing experiments (4C-seq and PE-4Cseq). We have shown that this polymer physics based pipeline accurately quantifies changes in chromatin interaction between distinct 4C experiments; importantly, the pipeline can be used not only to analyze allelic chromatin interactions through PE sequencing, but also perform standard interaction analyses using single end sequencing on 4C-seq experiments. The pipeline corrects for intrinsic biases in 4C sequencing data, normalizes captures, and extracts the contact probability profiles (CPP) of multiple viewpoints to perform quantitative comparisons between different samples.

Compared to statistical methods, our analysis pipeline utilizes a minimal number of 4 viewpoints for data normalization. Therefore, it can be extended to the analysis of Hi-C data [18], where every restriction fragment in the genome acts as a viewpoint (Mukhopadhyay S. et al., in preparation). Analyzing a number of 4C viewpoints systematically is a cheaper alternative to genome-wide (Hi-C) experiments; we showed that our pipeline can quantify signatures of higher-order chromosome organization similar to Hi-C, but in an allele-specific manner.

The newly developed pipeline is especially suited for the analysis of chromatin interaction changes upon copy number variation. CNVs can alter chromosome structure by introducing additional large pieces of DNA into specific regions (duplications), or by positioning two different chromatin regions near to each other (deletions). Therefore, it is important to distinguish interactions that arise from a clear biological source, from those originated by polymer entropy. Since we explicitly constructed a null model of chromatin CPP, we were able to distinguish specific interactions not explained by altered genomic proximity upon DNA deletion.

To test the utility of this new pipeline, we studied the changes in chromatin organization occurring upon the deletion of a 4.3 Mb segment of mouse chromosome region 4E2. The mouse 4E2 chromosome band is a particularly interesting region as it is orthologous to human 1p36, where heterozygous deletions are recurrent in cancer [29] and originate a mental retardation syndrome (Monosomy 1p36) [30, 41, 42]. To our knowledge, this is the first study of the effects on chromatin architecture of a recurrent human CNV modeled in mouse.

Using allele-specific PE-4Cseq experiments on heterozygote deletion/WT (*Df/*+ _*D*_^*Bl*6^) MEFs, we uncovered extensive changes in local and long-range chromatin interactions occurring upon the 4.3 Mb deletion in 4E2. Up to 22 % of chromosome 4 sequence in *Df*, and ~10 % of sequence in + _*D*_^*Bl*6^ are engaged in altered intra-chromosomal contacts (DIRs) as revealed through the analysis of 4 viewpoints bordering the deletion. ~9 Mb of sequence is shared between *Df* and + _*D*_^*Bl*6^ DIRs, implying that the 4.3 Mb deletion was not affecting *Df* chromatin conformation alone, but also + _*D*_^*Bl*6^ by *trans* mechanisms. Interestingly, both *Df* and + _*D*_^*Bl*6^ DIRs are enriched for CTCF and Smc1 binding; these proteins have been previously identified as structural determinants of chromosome structure [20–26]. While unique *Df* DIRs may be a direct consequence of the alteration of preferred chromatin conformation in the 4E2 region, future studies are needed to determine how *cis* and *trans* effects in chromatin organization are derived. Possible causes may be due to changes in the transcriptome owing to the deletion of genes inside the deletion. It is noteworthy to mention that gene expression analysis through RNA-seq revealed an upregulation in CTCF and other chromosome condensation genes in *Df/*+ _*D*_^*Bl*6^ MEFs compared to +^*129*^/+^*Bl6*^. The increased expression of these proteins in *Df/*+ _*D*_^*Bl*6^ MEFs could have important consequences in chromosome architecture for this genotype, and directly participate in DIR formation in *cis* and *trans*. Future chromatin immunoprecipitation experiments would be able to test this hypothesis.

A surprising reduction of local chromatin compaction was identified for viewpoints located near the telomeric end of the *Df* chromosome. This result points to a new higher-order architectural change upon the occurrence of the 4.3 Mb deletion affecting over 1 Mb of the terminal part of *Df*. Additional experiments will test the hypothesis of whether chromatin tethering points exist within or neighboring the deletion region (such as LADs), which could cause the intervening chromatin to extend upon the occurrence of the 4.3 Mb deletion. We verified a select few *Df* DIRs as well as a chromatin decompaction region through 3D DNA FISH. Notably, we found a strong agreement between the strengths and direction of change for both experimental modalities, thus validating our PE-4Cseq analysis pipeline and demonstrating the existence of chromatin reorganization upon the occurrence of the deletion in *Df*.

Altered gene expression, as measured by RNA-seq, was observed for a significant number of genes falling within or flanking *Df* and + _*D*_^*Bl*6^ DIRs. Interestingly, there is a high genome-wide correlation between allelic fold expression changes for the non-deleted alleles. While it is possible that chromatin interaction changes may be responsible for the altered expression of these genes, deriving associations between gene expression and chromatin conformation in a DNA deletion context is not easily attained.

Future studies of chromatin architecture in deletion/duplication model systems will be essential to expand our understanding of human disorders linked to the presence of CNVs or other genomic rearrangements such as balanced chromosome translocations. The *Df/*+ _*D*_^*Bl*6^ mouse genotype can be a model of heterozygous Monosomy 1p36 deletions in human. Such deletions frequently occur *de novo* and tend to have different sizes and positions [42–45]. Interestingly, a case of two individuals presenting similar clinical features and different deletion sizes and positions was reported [43], which suggests that Monosomy 1p36 could be a syndrome where deletions could have positional effects in addition to altering gene dosage. Examining various Monosomy 1p36 candidate genes (Additional file 13: Table S12), we discovered that most of their mouse orthologues fall within *Df/*+ _*D*_^*Bl*6^ DIRs. One such genes, endothelin-converting enzyme 1 (*Ece1*) [46, 47], 12 Mb upstream of the deletion region, is included in a *Df* DIR with a 30–40 % decrease in contact probability, and its expression is decreased in *Df/*+ _*D*_^*Bl*6^ MEFs. It is tempting to speculate that *Ece1* could be a candidate gene for which positional long-range effects on deletion alter gene expression, leading to phenotypic consequences in Monosomy 1p36 patients. However, this hypothesis remains to be tested.

## Conclusions

In summary, the development and validation of a new PE-4Cseq analysis framework based on polymer physics has revealed the extensive impact of a 4.3 Mb deletion in mouse chromosome 4 on local and global chromatin organization and gene expression. Future studies will assess deletions or duplications in other regions of the genome to determine if similar or different effects on gene expression and nuclear organization occur. Such studies will shed more light on the complex interplay between gene expression, chromosome structure and genomic rearrangements, and their impact on disease states.

## Methods

### 4E2 4.3 Mb deletion mouse models

In order to study chromatin organization of a DNA deletion in a heterozygous setting, we used an engineered mouse model for which we could identify the deletion chromosome from its WT homologue. For this reason, we used *Df*, a well characterized 4.3 Mb engineered deletion of the 4E band spanning D4Mit190-D4Mit51 derived from 129S5/SvEv^Brd^ ES cells [19]. First generation chimeras were bred and mated with C57BL/6J females to produce F1 *Df/*+ _*D*_^*Bl*6^progeny. F1 + ^*129*^/+^*Bl6*^ crosses were used as controls. MEFs were derived from 13.5 day *Df/*+ _*D*_^*Bl*6^and +^*129*^/+^*Bl6*^ embryos, and confluent passage 4 MEFs were used for all the experiments (see SI Experimental Methods for protocol details). The phenotypic characteristics of *Df/*+ _*D*_^*Bl*6^ MEFs and mice are as previously described [19].

### 4C template preparation

4C templates for *Df/*+ _*D*_^*Bl*6^ (MEF lines 129S5E71 and 129S5E98) and +^*129*^/+^*Bl6*^ (MEF lines 129S5E117 and 129S5E118) were prepared as described in [9]. See the Supplemental text for a detailed protocol on 4C template preparation, PE-4Cseq viewpoint sequencing, and reads mapping.

### Polymer physics analysis of PE-4CSeq data

Our generalized Gaussian model is defined by the joint distribution of the set of spatial positions {*x*_*i*_} of all the fragments *i*, and is given by $\boldsymbol{P}\left(\left\{{x}_i\right\}\right)={\displaystyle {\prod}_{i=1}^{N-1}}{\left(\frac{k_{i,i+1}}{2\pi}\right)}^{\frac{3}{2}} \exp \left[ - {k}_{i,i+1}{\left({x}_i-{x}_{i+1}\right)}^2/2\right],$ where *k*_*i*,*i* + 1_ is the spring constant associated with the chromatin segment intervening the centers of fragments *i* and *i* + 1 neighboring along the chromatin fiber. In a purely Gaussian model, the *k*_*i*,*i* + 1_ ∼ 1/*s*_*i*, *i* + 1_, where *s*_*i*, *i* + 1_ is the length of the chromatin segment. We allow for a general and local scaling of the contact probability, ${P}_{i,i+1}\sim {s}_{i,\ i+1}^{\nu_i},$ and therefore a general scaling of ${k}_{i,i+1}\sim {s}_{i,\ i+1}^{2{\nu}_i/3}$. A similar generalized Gaussian model, with no local variability of the exponent, is rather old in the polymer physics literature where it was studied as an approximation to the self-avoiding polymer [17]. The model offers an approximate description of the chromatin polymer at the length-scales of 4C measurements, which we discuss next.

We did not assume that the chromatin can be modelled at all length scales by a Gaussian polymer. The length-scales at which the model aims to describe the chromatin polymer are the typical distances between the multiple viewpoints; at around 0.5 Mb for our experiments. *Dpn*II cutter fragment sizes have a median of ~3Kb, which is the resolution at which each PE-4Cseq experiment queries the BCP (biased contact probability, see main text). However, at this resolution we only had “one-sided” BCP corresponding to one viewpoint with all fragments (*F*_*Ij*_ for viewpoint *I* and fragment *j*). In order to correct for biases in a general manner, the “two-sided” information of fragment-fragment contact is needed. We obtained this information in two steps: (1) We used the scaling form ${F}_{Ij}\sim {s}_{Ij}^{\nu_I}$ to fit the logarithm of BCP *F*_*Ij *_against the logarithm of genomic distances *s*_*Ij*_ (of viewpoint *I* to fragment *j*) using a smoothing spline. A smoothing spline, as opposed to a linear fit, is used because the scaling form is not expected to apply at very large separations (>100 Mb), where contacts are expected to be very rare and the signal to noisy ratio very poor, and nuclear confinement effects may dominate. The scaling form also breaks down at short distances corresponding to the measurement resolution we discussed above [16, 18]. The slope of the smoothing spline at 100 Kb is reported as the local exponent *ν*_*I*_. This length scale is chosen from observation that the spline fit is typically linear from 10 Kb to 10 Mb for all viewpoints (see Additional file 1). (2) The smoothing spline fit provides the average BCP *F*_*IJ*_ for viewpoints *I*with all other viewpoints *J*. It is important to consider the average, from such a log-log fit, instead of capture data for the fragments that overlap the viewpoint region *J*, because the latter may be dominated by specific biological interactions and is not the typical behavior of the null model. As mentioned in the text, *F*_*IJ*_ ≠ *F*_*JI*_ owing to biases.

We modeled biases by expressing *F*_*IJ*_ = *C*_*I*_*C*_*J*_*K*_*I*_ *P*_*IJ*_. Here *P*_*IJ*_ is the unbiased contact probability, given by our null model to be ${P}_{IJ}={s}_{Ij}^{\nu_I}$. The constants *C*_*I*_ and *C*_*J*_ are the viewpoint-dependent bias factors, and the constant *K*_*I*_ is the bias factor for the capture data experiment of viewpoint *I*. Note that the experiment for viewpoint *J* has a distinct bias factor, *F*_*JI*_ = *C*_*I*_*C*_*J*_*K*_*J*_ *P*_*IJ*_. In log space, a system of linear equations for *C*’s and *K*’s is solved by the least-square method. We only considered the nearest and next-neighbor viewpoint pairs in the system of equations and show that the biases for all other pairs are robustly reduced (Additional file 1: Figure S2).

To compute the CPP (Contact Probability Profile) we thresholded the capture reads for all fragments *α* with a cutoff of 5. We add a pseudo-count of 1 to all reads so that missing capture data do not give rise to false DIRs. The CPP for viewpoint *I* and fragment *α* is computed as ${P}_{I\alpha }=\frac{F_{I\alpha }}{C_I{K}_I},$ where *F*_*Iα*_ is the corresponding BCP. We smoothed the raw CPP using a Gaussian kernel with a standard deviation of 20 Kb (and window size four times the standard deviation). This smoothing factor is in the upper range of viewpoint sizes (~0.5–20Kb) which determine the genomic resolution of the PE-4Cseq measurement. We compared the CPPs to report DRCP (Differential Relative Contact Profile) at a signal strength (>5 %) defined by the percentage relative change for smoothed logarithm of CPP profiles of *a* and *b* being compared, (log *P*_*a*_ − log P_b_)/log *P*_*a*_. We then tested the statistical significance of each DRCP signal by comparing the raw data (CPPs) for *a* and *b* within a window of 80 Kb bracketing each DRCP location. Because the comparison is in difference of the mean of two samples we use a nonparametric test, the Mann-Witney *U* test. Only when the signal is statistically significant (*p*-value < 0.5) in *both* biological replicates in the window, we report the region as a significant DIR. The PE-4Cseq data discussed in this publication have been deposited in NCBI’s Gene Expression Omnibusand are accessible through GEO Series accession number GSE64360 (http://www.ncbi.nlm.nih.gov/geo/query/acc.cgi?acc=GSE64360).

### 3D DNA FISH

Pairs of differentially labeled (Alexa 594 and Alexa 488) BAC probes bordering the deletion start and end were used (Additional file 8: Table S7A). A third BAC probe (labeled with Alexa 647) was included inside the deletion region, so that each measurement is identified as belonging to either the WT or deletion chromosome based on signal proximity. *Df/*+ _*D*_^*Bl*6^ (129S5E56) and +^*129*^/+^*Bl6*^ (129S5E118) MEF slides were prepared using 60 % confluent cultures, and processed with a FISH protocol that preserves nuclear morphology [48]. Hybridization mixes were made by combining 3 μl nick-translated BAC probes (Abbott Molecular) with 5 μl mouse Cot1 DNA, 5 μl yeast tRNA, and 5 μl ssDNA, and lyophilized in Speed-Vac for ~20 min. Lyophilized probes were resuspended in 10 μl deionized formamide (Ambion), mixed with 10 μl 2X hybridization buffer, and dotted on clean slides. Coverslips of the specific analyzed genotypes were overlaid, and sealed with rubber cement. Sealed slides were put onto a 75 °C heat block for exactly 3 min, and hybridized overnight at 42 °C in humid chamber. Post-hybridization washes include: twice in 50 % formamide/2X SSC for 10 min at 42 °C (water bath), twice in 2X SSC for 10 min at 42 °C (shaking), twice in 1X SSC for 10 min at 42 °C (shaking). Coverslips were equilibrated in 4X SSC for 3 min at RT, stained with DAPI/4X SSC for 3 min, rinsed in 4X SSC, and mounted on clean microscope slides. Images from 100+ cells were obtained per BAC pair per genotype using an Applied Precision DeltaVision Core wide-field fluorescence microscope system (GE Healthcare) using a PlanApo 60X 1.40 numerical aperture objective lens (Olympus America). Image stacks were taken at 0.3 μm intervals throughout the entire cell and deconvolved using Applied Precision softWoRx software version 4.2.1 with default parameters. To measure query probe physical distances in 3D DNA FISH experiments we developed an automatic image analysis approach comprising 3D segmentation of cell nuclei and FISH signals, identification of relevant signals, automatic classification in WT or deletion chromosomes, and automatic 3D quantification (available upon reader’s request).

### Polymer physics analysis of FISH data

We used our generalized Gaussian model, introduced in the previous section, to fit the measured distributions of spatial separations of probe pairs in the FISH experiments. In this context, the model dictates the functional form of the probability distribution of spatial distances between FISH probes *I* and *J* in over a hundred nuclei; $P(r)dr = 4\ \pi\ {r}^2{\left(\frac{k_{IJ}}{2\ \pi}\right)}^{\frac{3}{2}} \exp \left[ - {k}_{IJ}\ {r}^2/2\right]\ dr,$ where *k*_*IJ*_ is the only fitting parameter. We observed that the model fits the measured distribution satisfactorily for all pairs [49]. A Gaussian model is the most parsimonious when quantifying differences in the mean behavior of probe-distances. The spring constant *k*_*IJ*_ is a quantitative measure of the strength of long-range chromatin interactions. In the FISH experiments, the homologous chromosomes in WT (+^*129*^ and + ^*Bl6*^) cannot be distinguished, however, the homologous chromosomes in the deletion heterozygote genotype (*Df* and + _*D*_^*Bl*6^) can be differentiated, due to one FISH probe being within the deletion region. We constructed analogous 4C comparisons, namely DRCP between *Df* and the average CP of +^*129*^ and + ^*Bl6*^. We reported the differential in *k*_*IJ*_ relative to the WT, (*k*_*IJ*_^*df*^ − *k*_*IJ*_^*WT*^)/*k*_*IJ*_^*WT*^, for quantitative comparison between PE-4CSeq results and FISH validation. We could have also reported probability of contacts between the BACset loci using the distribution of physical probe distances as measured on the microscope. However, the interaction volume within which two fragments are measured as a contact in the 4C capture data is unknown (i.e., the volumes of the nuclei from which the 4C template was obtained is unknown). A direct comparison between FISH and 4C is not feasible without the knowledge of this interaction volume.

### RNA-seq analysis

RNA from seven independent primary MEF lines was isolated (+^*129*^/+^*Bl6*^: 129S5E88, 129S5E90, 129S5E95; *Df/* + _*D*_^*Bl*6^: 129S5E36, 129S5E56, 129S5E71, 129S5E98) using TRIzol reagent (Ambion), and polyA+ RNA was isolated (Oligotex kit; QIAGEN). Stranded libraries were prepared using a protocol adapted for paired-end sequencing on the Illumina GA IIx platform [50]. PEx100 reads were separately aligned to both the C57BL/6J and 129S5/SvEv^Brd^ transcriptomes. We used the Ensembl release 72 gene set for the C57BL6/J transcriptome (GRCm38/mm10) [51]. The 129S5/SvEv^Brd^ transcriptome was constructed by modifying the C57BL/6J transcriptome using SNPs and indels calls derived from [31]. Where multiple transcripts exist for a gene, we selected the longest transcript as the representative transcript for the gene in the transcriptome. We used the GSNAP alignment algorithm with the parameter of no mismatches [52]. Reads were filtered to keep only those with one best mapping location. To obtain estimates of expression values, we only counted those reads aligning at a gene location if both reads of a paired-end set were mapped to the same gene. To avoid biological interpretation from mapping noise, we excluded genes with less than 10 reads mapping to each allele if this occurs across genotypes. Differential expression analyses were performed using the R Bioconductor package – DESeq [53], using an FDR cut-off of 0.05. We performed non-allele-specific differential expression analyses (pairwise between WT and deletion heterozygote) using counts summed from both alleles. Allele-specific analysis were performed only using reads that mapped to the transcriptome of each strain and compared in a pairwise manner, that is, between *+*^*129*^*/+*^*Bl6*^ samples (C57BL/6J x 129S5/SvEv^Brd^) and *Df/*+ _*D*_^*Bl*6^ samples. To account for the allelic mapping biases that is a result of more reads mapping to the C57BL/6J transcriptome, we tested for changes in the proportion of reads mapping to each allele between treatment and WT groups, on a gene by gene basis, to determine whether similar degree of changes to expression levels occurred between alleles. Counts were normalized using DESeq and tests were done using the R function, prop.test, using median counts across replicates and *p*-values were adjusted for multiple testing in R using the fdr method (adjusted *p*-value cut-off = 0.01). The software GREAT was used for functional term enrichment analysis with single gene associations [54] as well as WEB-based GEne SeT AnaLysis Toolkit with hypergeometric tests and Bonferroni corrections [55]. Locations were mapped to the NCBI37/mm9 genome for correlation testing using UCSC LiftOver. The RNA-Seq data discussed in this publication have been deposited in NCBI’s Gene Expression Omnibus (Zepeda Mendoza et al., 2015) and are accessible through GEO Series accession number GSE64360 (http://www.ncbi.nlm.nih.gov/geo/query/acc.cgi?acc=GSE64360).

## Additional files

Additional file 1: Figure S1. Result of bias-correction for a simulation of biases in multi-viewpoint 4C experiment (with 100 viewpoints). **Figure S2.** Overview of PE-4Cseq methodology. **Figure S3.** Positions of DIRs for all viewpoints 1, 2, 11 and 12 are compared against structural protein binding sites. First track is for *Df* and +^*129*^ comparisons and second track is for + _*D*_^*Bl*6^ and + ^*Bl6*^ comparison. **Figure S4.** Calculated *ν* per viewpoint for + _*D*_^*Bl*6^ vs + ^*Bl6*^. + _*D*_^*Bl*6^ (red squares). **Figure S5.** Distance distributions between query probes for (A) BACset1, (B) BACset2, (C) BACset3, (D) BACset4, FISH experiments. (DOCX 1390 kb) Additional file 2: Table S1. (a) Positions and (b) C57Bl6/J-129S5/SvEvBrd SNP information for analyzed PE-4Cseq viewpoints on chromosome 4. Primers used for Sanger sequencing are shown. (XLSX 26 kb) Additional file 3: Table S2. Summary of median magnitude of change, direction, and number of *df* DIRs for viewpoints 1, 2, 11, and 12. Notice how viewpoint 1 displays an increase in contact probabilities with surrounding sequences (64 % of total detected regions), while viewpoints 2 and 11 show mostly a decrease in interactions (60 % for viewpoint 2, and 95 % for viewpoint 11). Viewpoint 12 has both increase and decrease in interactions in approximately the same magnitude (~50 %). (XLSX 10 kb) Additional file 4: Table S3. Summary of median magnitude of change, direction, and number of + _*D*_^*Bl*6^ DIRs for viewpoints 1–12. Notice how only viewpoint 5 displays an increase in contact probabilities (63 % of total regions). Viewpoints 3, 4, and 9 show a ~50 % split between regions with an increase and decrease in contact probabilities, while the rest of the viewpoints show a variable range in the decrease in interactions (60–79 % of total regions per viewpoint). (XLSX 10 kb) Additional file 5: Table S4. Unique (a) *df* and (b) *Df/*+ _*D*_^*Bl*6^ regions. All chromosome 4 positions are shown. (XLSX 12 kb) Additional file 6: Table S5. (a) Summary of *df* DIRs overlap with CTCF, Mediator, and cohesin binding sites. Column 1, *Region*, refers to the viewpoint assessed. Column 2, *diffsites*, refers to *df* DIRs. Column 4, *no. sites* and feature name corresponds to the number of DIRs that contain the specified genomic feature. Column 6, *bp sites* feature, presents the sum of DIRs bp which contain the specified feature. Column 8, *no.* features, indicates the number of features included inside DIRs. Percentages in columns 5, 7, and 9 are calculated based on the total number of regions or features in the preceding column. (b) Summary of *df* DIRs overlap with CTCF, Mediator, and cohesin binding sites. Column 1, *Region*, refers to the viewpoint assessed. Column 2, *diffsites*, refers to *df* DIRs. Column 4, *no. sites* and feature name corresponds to the number of DIRs that contain the specified genomic feature. Column 6, *bp sites* feature, presents the sum of DIRs bp which contain the specified feature. Column 8, *no.* features, indicates the number of features included inside the DIRs. Percentages in columns 5, 7, and 9 are calculated based on the total number of regions or features in the preceding column. (c) Summary of + _*D*_^*Bl*6^ DIRs overlap for viewpoints 1, 2, 11, and 12 with CTCF, Mediator, and Smc1 binding sites. Column identities are as described in (a). (d) Summary of Monte Carlo simulations for assessing statistical significance of protein binding overlaps for + _*D*_^*Bl*6^ DIRs for viewpoints 1, 2, 11, and 12. Column identities are as described in (b). Notice the significant *p*-values obtained for CTCF and Smc1 binding (*p*-val < 0.001, rounded down to zero in table). (XLSX 31 kb) Additional file 7: Table S6. Sequencing LAD (sLAD) positions detected for the terminal part of chromosome 4. Data derived from 3 T3 MEFs^40^. Marked in yellow is the extensive sequence stretch bordering the CNV devoid of sLADs. Marked in green are the sLAD regions inside the CNV. (XLSX 11 kb) Additional file 8: Table S7. BACS used for selected PE-4Cseq and chromatin decompaction regions. a) Location of BACs used in 3D DNA FISH experiments for the validation of df PE-4Cseq DIRs and chromatin decompaction. b) Number of CTCF, Med1, Med12, and Smc1 protein binding sites^33^ overlapping each BAC used for 3D DNA FISH experiments. c) Proportions of CTCF, Med1, Med12, and Smc1^33^ protein binding sites overlapping each BAC used for 3D DNA FISH experiments normalized by BAC size. (XLSX 377 kb) Additional file 9: Table S8. Combined DE genes of three +^*129*^/+^*Bl6*^ and four *Df/*+ _*D*_^*Bl*6^ MEF revealed by RNA-Seq. (XLSX 170 kb) Additional file 10: Table S9. GO “condensed nuclear chromosome” table of DE genes in *df/+* ^*Bl6*^ MEFs. (XLSX 12 kb) Additional file 11: Table S10. 129S5/SvEvBrd allele-specific DE genes of three +^*129*^/+^*Bl6*^ and four *Df/*+ _*D*_^*Bl*6^ MEF revealed by RNA-Seq. (XLSX 40 kb) Additional file 12: Table S11. C57Bl6/J allele-specific DE genes of three +^*129*^/+^*Bl6*^ and four *Df/*+ _*D*_^*Bl*6^ MEF revealed by RNA-Seq. (XLSX 48 kb) Additional file 13: Table S12. Candidate genes associated with different Monosomy 1p36 phenotypes. Their corresponding mouse homologues are shown in column 3, together with their chromosomal positions (columns 5,6). Their overlaps with *df* DIRs are displayed in columns 7–9, as well as the direction of change of the contact probabilities (1 = increase,–1 = decrease. Both compared to +^*129*^). RNA-Seq derived expression in *Df/+* ^*Bl6*^ MEFs is shown in columns 11,12. Mouse gene coordinates are expressed in NCBI37/mm9 assembly, while human is GRCh38. (XLSX 11 kb)
