# Supplementary material for: Quantitative analysis of chromatin interaction changes upon a 4.3 Mb deletion at mouse 4E2
Source: BMC Genomics. 2015 Nov 21;16:982. doi: 10.1186/s12864-015-2137-5 (PMC4654810; doi:10.1186/s12864-015-2137-5)
Supplement: Additional file 1: Figure S1. — Result of bias-correction for a simulation of biases in multi-viewpoint 4C experiment (with 100 viewpoints). Figure S2. Overview of PE-4Cseq methodology. Figure S3. Positions of DIRs for all viewpoints 1, 2, 11 and 12 are compared against structural protein binding sites. First track is for Df and +129 comparisons and second track is for + DBl6 and + Bl6 comparison. Figure S4. Calculated ν per viewpoint for + DBl6 vs + Bl6. + DBl6 (red squares). Figure S5. Distance distributions between query probes for (A) BACset1, (B) BACset2, (C) BACset3, (D) BACset4, FISH experiments. (DOCX 1390 kb) [file 12864_2015_2137_MOESM1_ESM.docx]

**Supplementary Information**

**Quantitative analysis of chromatin interaction changes upon a 4.3Mb deletion at mouse 4E2**

Cinthya J. Zepeda-Mendoza*^1^, Swagatam Mukhopadhyay*^2^, Emily S. Wong^3^, Nathalie Harder^4^, Erik Splinter^5^, Elzo de Wit^5^, Melanie A. Eckersley-Maslin^1,6^, Thomas Ried^7^, Roland Eils^4^, Karl Rohr^4^, Alea Mills^1,2^, Wouter de Laat^5^, Paul Flicek^3^, Anirvan M. Sengupta^8^, David L. Spector^1,2^

* These authors contributed equally to this work.

^1^Watson School of Biological Sciences, Cold Spring Harbor Laboratory. 1 Bungtown Road, Cold Spring Harbor, New York. 11724. USA.

^2^Cold Spring Harbor Laboratory. 1 Bungtown Road, Cold Spring Harbor, New York. 11724. USA

^3^ The European Molecular Biology Laboratory, European Bioinformatics Institute. Wellcome Trust Genome Campus. Hinxton, Cambridge. CB10 1SD, UK.

^4^University of Heidelberg, BioQuant, IPMB, and German Cancer Research Center (DKFZ), Dept. Bioinformatics and Functional Genomics, Biomedical Computer Vision Group. Im Neuenheimer Feld 267, 69120 Heidelberg. Germany.

^5^Hubrecht Institute-KNAW & University Medical Center Utrecht, Uppsalalaan 8, 3584 CT Utrecht, The Netherlands.

^6^Epigenetics Programme, Babraham Institute. Babraham Research Campus. Cambridge, CB22 3AT. UK.

^7^Center for Cancer Research. National Cancer Institute. National Institutes of Health. 50 South Drive, Bldg. 50, Rm. 1408. Bethesda, Maryland. 20892. USA.

^8^Department of Physics and Astronomy. Rutgers, the State University of New Jersey. 136 Frelinghuysen Road. Piscataway, New Jersey. 08854-8019. USA.

Correspondence should be addressed to S.M ([swagatam.mukhopadhyay@gmail.com](mailto:swagatam.mukhopadhyay@gmail.com)) and D.L.S. ([spector@cshl.edu](mailto:spector@cshl.edu))

**Contents**

**Supplementary methods** 3

Generation of F1 +129/+Bl6 *and Df*/$+DBl6$ *Embryos* 3

MEF Preparation and Cell Culture 3

3D DNA FISH 3

4C Template Preparation 4

PE-4CSeq Viewpoint Amplifications, Sequencing, and Reads Mapping 5

Simulation study of bias correction for PE-4CSeq data 6

Random sampling overlaps for DIR and structural proteins binding sites 6

**Supplemental Figures** 8

Figure S1 8

Figure S2 10

Figure S3 11

Figure S4 12

Figure S5 13

**Supplementary Tables** 17

Table S1. 17

Table S2. 17

Table S3. 17

Table S4. 17

Table S5. 17

Table S6. 17

Table S7. 17

Table S8. 18

Table S9. 18

Table S10. 18

Table S11. 18

**References** 18

# **Supplementary methods**

## **Generation of F1 +129/+Bl6 and *Df*/**$\mathbf{+}_{\boldsymbol{D}}^{\boldsymbol{Bl}\mathbf{6}}$ **Embryos**

The *Df/Dp* mouse strain described in Bagchi et al, 2007, was re-established by injecting the chromosomally-engineered D4Mit190-D4Mit51 *Df/Dp* ES cell line into C57BL/6J blastocysts and put into surrogate mothers. F1 progeny from segregating chimeras X C57BL/6J crosses were genotyped by PCR on tail-derived DNA. *Df* genotyping was performed using primer pairs #2137 (df FWD): 5' – CCTCATGGACTAATTATGGAC – 3' and #2138 (df REV): 5' – CCAGTTTCACTAATGACACA – 3', using the following PCR conditions: 94°C for 4 minutes, followed by 40 cycles of 30 s at 94°C, 1:15min at 53°C, and 2:30min at 70°C, and a final cycle of 5 min at 70°C. 25μl genotyping reactions were made using 2.5 μl 10X PCR buffer, 0.5 μl of dNTPs (10mM, New England BioLabs), 1.25 μl of each primer (10μM), 1 μl of DNA (20-50ng), 1.25 μl of DMSO, 17.1 μl of dH2O, and 0.15 μl of TaqPolymerase (AmpliTaq DNA polymerase, Applied Biosystems, 5U/μl). PCR product is approximately 2.2Kb in size. dp genotyping was performed using primer pairs #1991 (dp FWD): 5' – CGGTAGAATTTCGAGGTCGCTAG - 3' and #1992 (dpREV): 5' – GCCCAAGCTGATCCGGAACCC – 3', using the following PCR conditions: 94°C for 4 minutes, followed by 40 cycles of 30 s at 94°C, 1 min at 63°C, and 2:30min at 70°C, and a final cycle of 5 min at 70°C. 25μl genotyping reactions were made using 2.5 μl 10X PCR buffer, 0.5 μl of dNTPs (10mM), 1.25 μl of each primer (10μM), 1 μl of DNA (20-50ng), 18.35 μl of dH2O, and 0.15 μl of TaqPolymerase (AmpliTaq DNA polymerase, Applied Biosystems, 5U/μl). PCR product is approximately 800bp in size.

## **MEF Preparation and Cell Culture**

Embryos of *Df/Dp* male chimeras X C57BL6/J crosses were dissected at 13.5 days after plug observation. Heads were removed for DNA extraction and genotyping, and the bodies minced by passing through syringes with 18G1 ½ and 20G 1 ½ needles and plated on 10cm dishes previously coated with 0.1% gelatin and using for culture Dulbecco's Modified Eagle Medium (DMEM) High Glucose (4.5g/L) supplemented with 10% Fetal Bovine Serum (FBS) (v/v), 50U/ml Penicillin G, and 100µg/ml Streptomycin sulfate. Cells were incubated on 5% CO_2_ at 37°C, and passaged every 2-3 days depending on confluency and growth rate. All experiments were subsequently performed on MEF plates 10 hours after reaching confluency at passage 4.

## **3D DNA FISH**

3D DNA FISH was performed as described in [^53^](#_ENREF_53). In summary, ~60% confluent MEF #1.5 22mm acid cleaned coverslips were prepared by fixing cells in 4% PFA/PBS for 10min at RT. During the last minute, 2 drops of 0.5% Tx100/PBS were added. Coverslips were then washed 3 times in PBS for 5 min at RT. Nuclei were permeabilized by incubating coverslips in 0.5% Tx100/PBS for 10 min at RT, washed in PBS 3 times for 5 min, and incubated with 0.1mg/ml RNAseA/PBS for 30 min at 37°C. After washing coverslips 3 times in PBS for 5 min at RT, these were transferred twice to coplin jars with freshly-made 20% Glycerol/PBS. Coverslips were incubated overnight at 4°C in 20% Glycerol/PBS, and subsequently submerged into liquid nitrogen, frozen and thawed for a total of 5 times, soaking with 20% Glycerol/PBS between each freeze/thaw cycle. Cells were washed 3 times in PBS for 5 min at RT, briefly rinsed in 0.1N HCl, and then incubated in fresh 0.1N HCl for 10 mins. Finally, coverslips were washed 3 times in PBS for 5 min at RT, equilibrated in 2x SSC for 5 min, and incubated in 50% Formamide/2x SSC for 30 min. Prepared coverslips were stored at 4°C until further used.

Hybridization mixes were prepared by nick translating isolated BAC DNA. Reactions include: x µl (2µg) of maxi-prep BAC DNA, 22-x µl nuclease free water, 2.5µl 0.2mM labeled dUTP (green: Alexa 488, red: Alexa 594; Cy-5: Alexa 647, Life Technologies), 5µl 0.1mM dTTP (Roche), 10µl dNTP mix (0.1mM, New England BioLabs), 5µl 10X nick translation buffer, and 5µl nick translation enzyme (Abbott Molecular Inc.). Reactions were incubated at 15°C for 10hrs, heat inactivated at 70°C for 10 min, and cooled down to 4°C. Reactions were transferred to 1.5ml tubes and mixed with 1µl of 0.5M EDTA, 1µl of linear acrylamide (Ambion), 5 µl of 3M NaOAc (pH 5.2), and 125 µl of 100% EtOH (-20°C cold), and incubated overnight at -20°C. Samples were then centrifuged at 20,000g for 1 hr at 4°C. At this point the colored pellet should be visible. Pellets were cleaned by adding 1ml of 75% EtOH, centrifuged at 14,000RPM for 5 min, repeating the last two steps, drying the pellet in 37°C incubator for 15 min, and dissolving in 50 µl of DEPC-treated water by vortexing at 37°C for 1 hr. Hybridization mixes were made by combining 3µl nick-translated probe with 5 µl mouse Cot1 DNA, 5 µl yeast tRNA, and 5 µl ssDNA, and lyophilized in Speed-Vac for ~20min. Hybridization buffer was made using 4XSSC, 20% dextran sulfate, and dH2O, mixed together and kept in the heating block at 37°C. Lyophilized probes were resuspended in 10µl formamide (Ambion) and kept shaking in the heating block at 37°C for at least 30min. 10µl of hybridization buffer were added to the 10µl fluorescent probes, and the mix was loaded onto clean glass slides. The prepared coverslips were mounted cell-side down onto the hybridization mix, sealed with rubber cement, and kept in the dark until dry. Sealed slides were put onto 75°C heat block for exactly 3min, and hybridized overnight at 42°C in humid chamber. Post-hybridization washes include: twice in 50% formamide/2x SSC for 10min at 42°C (water bath), twice in 2x SSC for 10min at 42°C (shaking), twice in 1x SSC for 10min at 42°C (shaking). Coverslips were equilibrated in 4x SSC for 3min at RT, stained with DAPI/4x SSC for 3min, rinsed in 4x SSC, and mounted on clean microscope slides. Coverslips were sealed with nail polish, and imaged using an Applied Precision DeltaVision Core wide-field fluorescence microscope system (GE Healthcare, Issaquah, WA) equipped with a PlanApo 60x 1.40 numerical aperture objective lens (Olympus America).

## **4C Template Preparation**

4C templates for *Df/*$+_{D}^{Bl6}$ (MEF lines 129S5E71 and 129S5E98) and +*^129^*/+*^Bl6^* (MEF lines 129S5E117 and 129S5E118) were prepared as described in [^12^](#_ENREF_12). Briefly, 1x107 MEFs were cross-linked for 10 min at RT using 2% formaldehyde (Calbiochem) and 10%FCS in PBS (pH 7.4). 10 ml reactions were transferred to ice and added 1.425ml of 1M glycine, followed by centrifugation for 8 min at 225g at 4°C. Supernatant was subsequently removed and the resulting cell pellet resuspended in 500 μl of ice cold nuclei buffer (10mM Tris pH 7.6, 10mM NaCl, 2mM MgCl2, dH2O) containing protease inhibitors (Roche) for 10 min on ice. An equal volume of nuclei buffer/0.5% NP-40 was added to the tube and incubated for 5 min on ice. Samples were vortexed for 10 s and centrifuged for 1 min at 1,000g and 4°C. Cells were washed once in nuclei buffer/0.5% NP-40 containing protease inhibitors and centrifuged for 1 min at 1,000g and 4°C.

Pellets were resuspended in 450µl dH2O and 60µl 10X restriction buffer (buffer 2 supplied with *Hind*III enzyme, New England BioLabs), incubated 1 hr with 15µl 10% SDS shaking at 900RPM at 37°C, and followed by an additional 1 hr incubation with 75µl 20% Triton X-100. 5 µl aliquots were taken as undigested controls and stored at 4°C. Samples were subsequently digested by adding 800U of *Hind*III (New England BioLabs) and incubating overnight at 37°C while shaking. 5 µl aliquots were taken as digested controls and de-crosslinked by incubation with 10 μl Proteinase K (10mg/ml, Roche) in 90 μl of 10 mM Tris (pH 7.5) at 65 °C for 1 h. Digestion efficiencies were estimated based on the pattern of smear of the undigested and digested controls by running 20 μl of decrosslinked sample on a 0.6% agarose gel. If digestion was sufficient, *Hind*III was inactivated by incubating the sample for 20 min at 65°C (shaking gently). The digested nuclei were transferred to a 50 ml falcon tube and mixed with 5.7ml dH2O, 700 μl 10X Ligase Buffer, and 50U T4 Ligase (Roche), and incubated overnight at 16°C. Ligation efficiency was determined by taking 100 μl of ligation reaction and incubating 1 hr at 65°C with 5 μl Proteinase K (10 mg/ml). When run in a 0.6% agarose gel, ligated DNA should appear as a single upper band similar to the undigested control. If ligation occurred, DNA crosslinks were reversed by adding 30 μl of 10 mg/ml Proteinase K and incubation at 65 °C overnight. Subsequently, 15 μl of 20 mg/ml PureLink RNase A (Invitrogen) was added and the reactions incubated for 45 min at 37 °C, followed by phenol extraction and DNA purification as described in Splinter et al, 2012. The DNA pellet was dissolved in 150 μl of 10 mM Tris (pH 7.5), and digested overnight with 50U *Dpn*II (New England BioLabs) at 37°C while shaking. An aliquot of 5 μl was taken from the *Dpn*II reaction and mixed with 95 μl of 10 mM Tris (pH 7.5), and 20 μl loaded into a 0.6% agarose gel to assess digestion efficiency. If sufficient digestion was achieved, *Dpn*II was heat inactivated by incubating 20 minutes at 65°C, and DNA was ligated at low concentrations (12.1ml dH2O, 1.3ml 10X ligation buffer, 100U T4 DNA Ligase) overnight at 16°C. DNA was phenol extracted and ethanol precipitated with glycogen (Roche) as a carrier. The resulting 4C templates were purified using QIAquick PCR purification kit columns (Qiagen), dissolved in 10 mM Tris (pH 7.5), and stored at -20°C.

## **PE-4CSeq Viewpoint Amplifications, Sequencing, and Reads Mapping**

Inverse 4C amplification primers were designed per viewpoint following standard rules for PCR primer design, and checking alignment uniqueness to the desired fragment as compared to the rest of the genome. Primers used in this study are listed in Supplementary Table 1c. Additionally, amplification primers for all viewpoints were added the PE1 and PE2 Illumina paired-end primers plus a 1-2 nucleotide barcode in their 5' ends for HiSeq PEx100 sequencing.

Each of the 14 viewpoints was amplified from the available *Df/*$+_{D}^{Bl6}$ and +*^129^*/+*^Bl6^* 4C templates in reactions using 3.2µg 4C template, 16µl dNTP (10mM, New England BioLabs), 24µl reading primer PE1 of a 1µg/µl primer stock, 24µl reading primer PE2 of a 1µg/µl primer stock, 11.2 µl Expand Long Template polymerase (Roche), 80µl 10X PCR buffer 1 (supplied with polymerase), and dH2O until completing 800µl total. This volume is then mixed and separated into 16x50µl PCR reactions, and run using the following program: 94 °C for 2 min, followed by 30 cycles of 15 s at 94 °C, 1 min at 55 °C and 3 min at 68 °C, and one final step of 5min at 68 °C. PCR reactions were subsequently collected and pooled together, and purified using the High Pure PCR Product Purification Kit (Roche) for viewpoints amplified on 129S5E71 and 129S5E117 4C templates, or using AMPure beads (Beckman Coulter) with a 0.9X volume ratio for viewpoints amplified from 129S5E98 and 129S5E118 4C templates. Equimolar amounts of isolated captured viewpoints were pooled together using the KK4824 kit to correct for insert size lengths (Kapa Biosystems). Pooled libraries were sequenced using two lanes of HiSeq PE100.

Obtained reads were separated using custom perl scripts based on the sample and genotyping SNP on PE1 reads. PE2 reads were trimmed to 30bp to have the highest quality bases for captures mapping. Alignments were performed using bowtie against a reduced database of sequences bordering *Hind*III restriction sites in the NCBI37/mm9 assembly. Up to 3 mismatches were accepted per read to account for SNPs in the 129S5/SvEvBrd sequence, and only uniquely mapped reads were taken into account for the 4C data analysis.

## **Simulation study of bias correction for PE-4CSeq data**

In order to determine the performance of the bias correction method perform the following test on simulated data. We first compute the contact probability $P\left( i,j \right)=\prod_{i=1}^{N-1} \left( \frac{k_{i,j}}{2\pi} \right)^{\frac{3}{2}}$ for a Gaussian polymer of $N$ fragments. The spring constants $k_{i,j}=\frac{1}{L_{i,j}}$, where $L_{ij}$ is the genomic separation between fragments. The simulated data is $F\left( i,j \right)=C_{i} C_{j}K_{i} P\left( i,j \right)$ where $C_{i}, K_{i}$ are uniform random numbers in $\left( 0,1 \right]$; $C_{i}$are the bias factors associated with each fragment, and $K_{i}$are the bias factors associated with each experiment (see main Paper, Methods). From the fall off (in log-scale) of $F\left( i,j \right)$, we determine that the polymer obeys random walk scaling. We then compute $P^{c}\left( i,j \right)$ from the Gaussian form, where the superscript indicates “computed”. We then solve the set of linear equations for the unknowns $C_{i}^{c}$ and $K_{i}^{c}$ and the computed $P^{c}(i,j)$. The equations are, $\log F\left( i,j \right)=\log C_{i}^{c}+\log C_{j}^{c}+ \log K_{i}^{c}+\log P^{c}\left( i,j \right)$ and $\log F\left( j,i \right)=\log C_{i}^{c}+\log C_{j}^{c}+ \log K_{j}^{c}+\log P^{c}\left( i,j \right)$. Note that the experiment bias is distinct. The fragment pairs considered are only the nearest and the next nearest neighbors $\left( i,j \right).$ The method succeeds in eliminating biases for the simulated data $F(i,j)$ of all other fragment pairs, see Supp. Fig. 1.

We have repeated the simulation for (uniform) non-random-walk fall-off exponent $\nu$. For the present work, the genomic separation between viewpoints are of the order of Mb, and we observed that assuming a uniform fall-off of $\nu= -1$suffices in bias correction. The case of locally varying scaling is beyond the scope of this paper and is discussed elsewhere (Mukhopadhyay *et al*., in preparation).

## **Random sampling overlaps for DIR and structural proteins binding sites**

To assess whether the CTCF, Smc1, Med1, and Med12 overlap ratios were significant for the *df* and $+_{D}^{Bl6}$ DIRs, we computed the probability of exceeding the number of protein binding sites in these regions against randomly chosen sequences of the same size as the analyzed DIRs. The intersections between all MEF-derived structural protein binding datasets[^33^](#_ENREF_33) and the DIRs was performed with custom-made perl scripts. The results from the intersections were subsequently used to establish the observed values against which random sampling was be compared. 1,000 random sampling repeats were computed using the BEDTools suite[^61^](#_ENREF_61) Shuffle program. New locations for each of the original DIRs were selected while preserving their size in chromosome 4. A p-value was derived by counting the number of times that the number of shuffled intersections exceeds the observed intersections. If 0, then p-val is less than 0.001.

# **Supplemental Figures**

## **Figure S1**

**(A)**


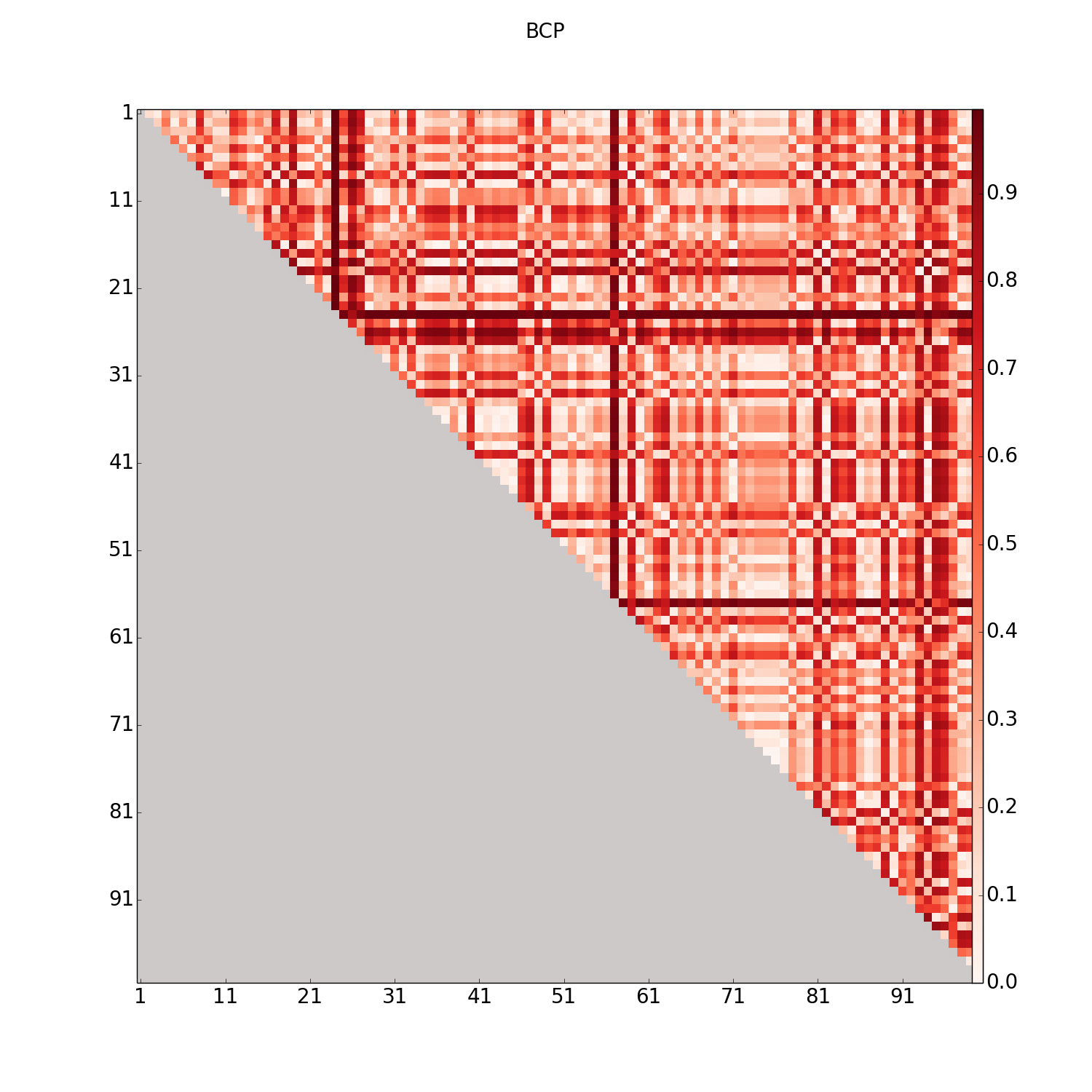


**(B)**

**
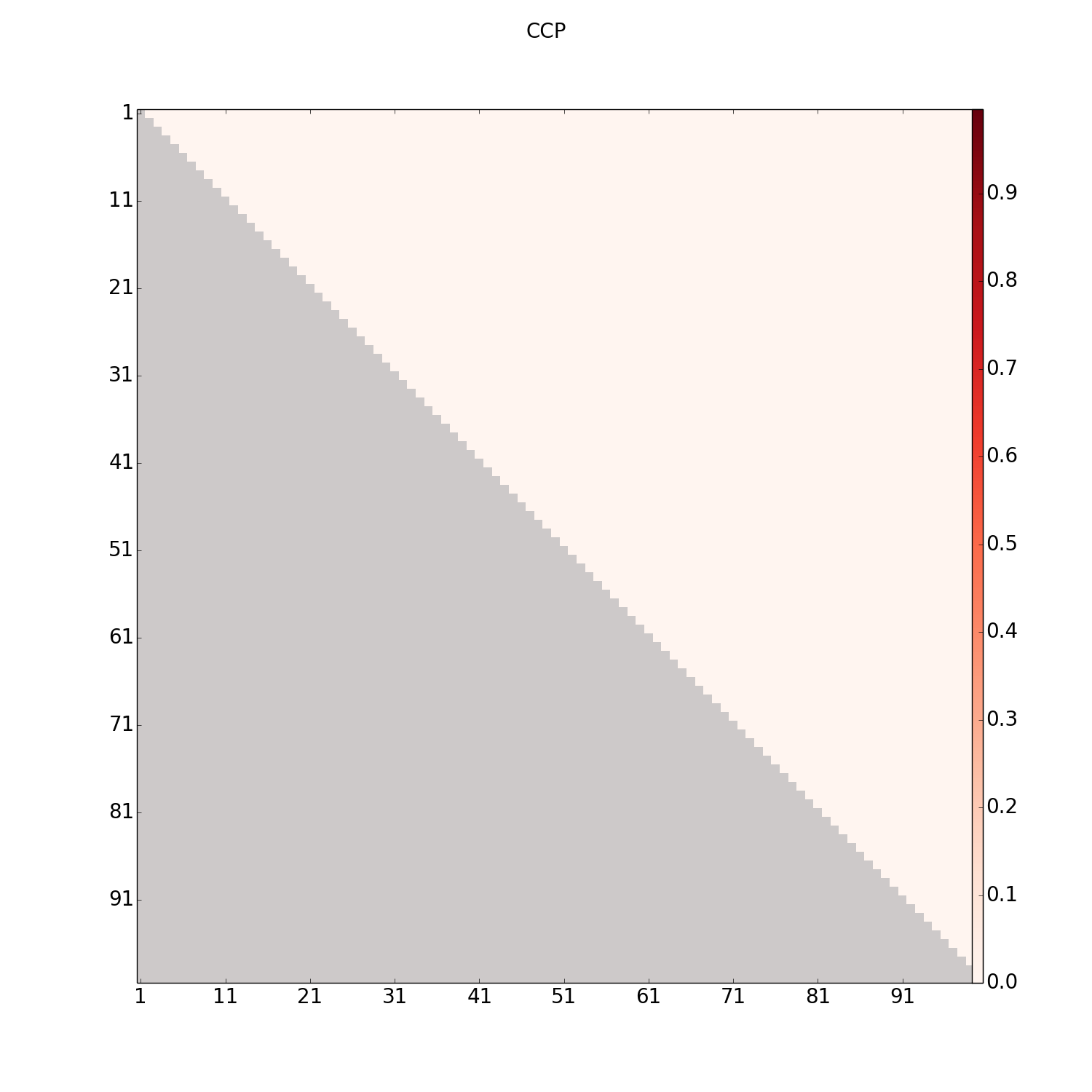
**

**Figure S1.** Result of bias-correction for a simulation of biases in multi-viewpoint 4C experiment (with 100 viewpoints). Viewpoints index is on the $x$and $y$axis. The heatmap in (A) is for the relative asymmetry matrix $\frac{\left| F_{ij}-F_{ji} \right|}{F_{ij}+F_{ji}}$ in BCP $F_{ij}$ where only the upper triangle is shown because the matrix is symmetric. The heatmap in (B) on the same scale is for the relative asymmetry $\frac{\left| P_{ij}-P_{ji} \right|}{P_{ij}+P_{ji}}$for $P_{ij}$ obtained after bias-correction. Notice the reduction in both row and column-wise biases and in the net asymmetry between viewpoints. Heatmaps are displayed in a log_10_ scale

## **Figure S2**

**
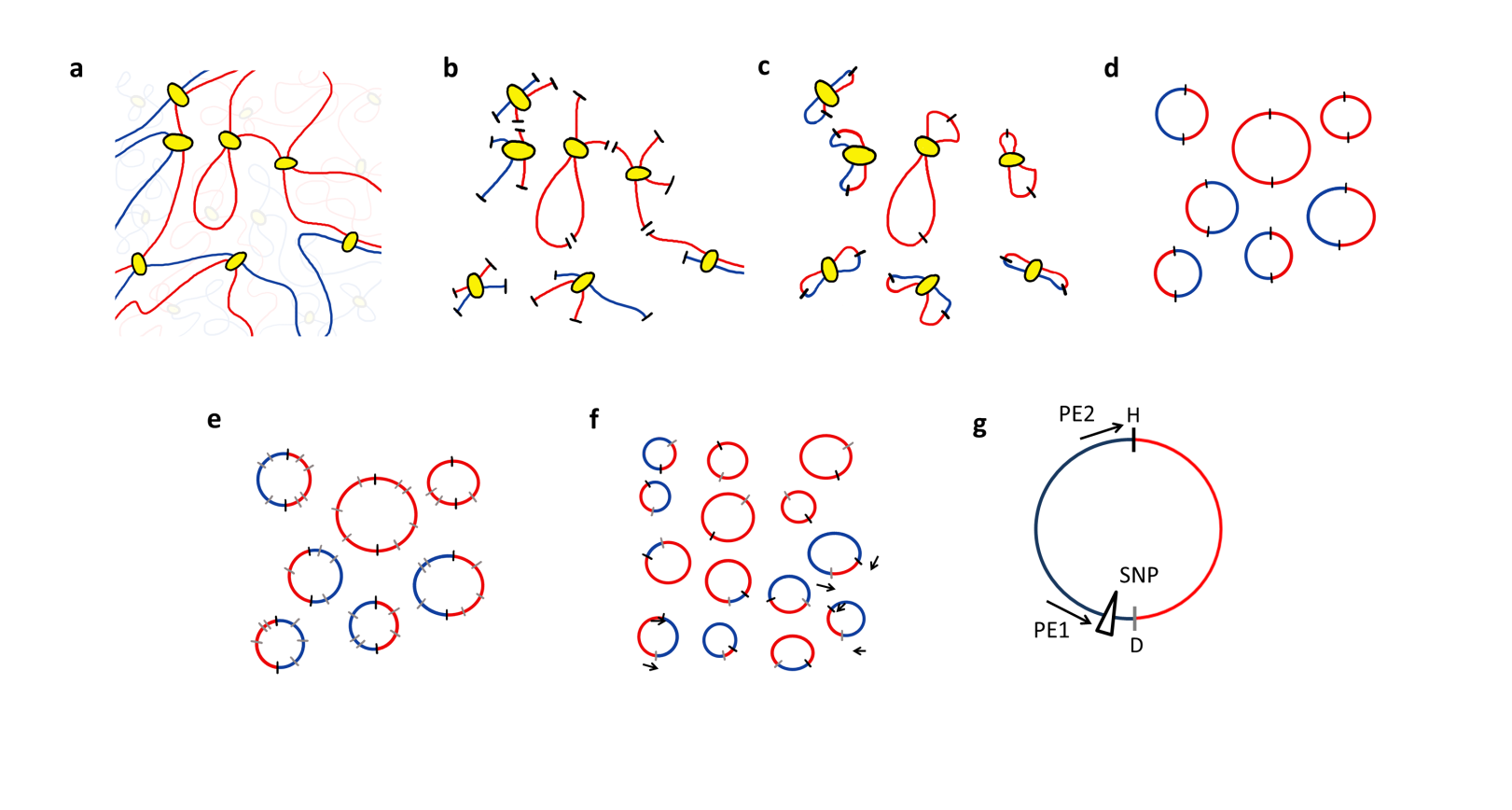
**

**Figure S2. Overview of PE-4Cseq methodology.** **a)** Chromatin is cross-linked inside nuclei with formaldehyde. Regions that were near to each other in the 3D space either by protein bridges (yellow dots), or by mere co-localization, will be fixed and captured. Most procedures use an average of ten million cells per experiments. **b)** Primary restriction enzyme digestion of crosslinked chromatin. This step is typically performed using a 6bp restriction cutter, such as *Hind*III (restriction sites marked in black lines). **c)** Ligation of digested fragments. This step is performed at very dilute concentrations to favor intra-molecular ligation of fragments. **d)** De-crosslinking and purification of 3C library. The final product of the 3C procedure is a library that represents all sequences that were in physical proximity in the original cell population. **e)** The 3C template is subjected to a second round of restriction digestion, typically with a 4bp cutter like *Dpn*II (shown in grey lines). **f)** Dilute ligation of cut DNA results in the generation of a 4C library, where template DNA circles are smaller compared to the 3C template. Interacting partners (red portion of the circle) of a region of interest (blue portion of the circle) are amplified using specific primers (black arrows) and their identities determined by either sequencing or DNA hybridization in microarrays. **g)** PE-4Cseq strategy outline. PE-4Cseq makes use of paired-end sequencing, in which one read (PE1) amplifies a genotyping SNP (white triangle) between different alleles, which is subsequently used to separate the interacting partner amplified in PE2 reads.

## **Figure S3**


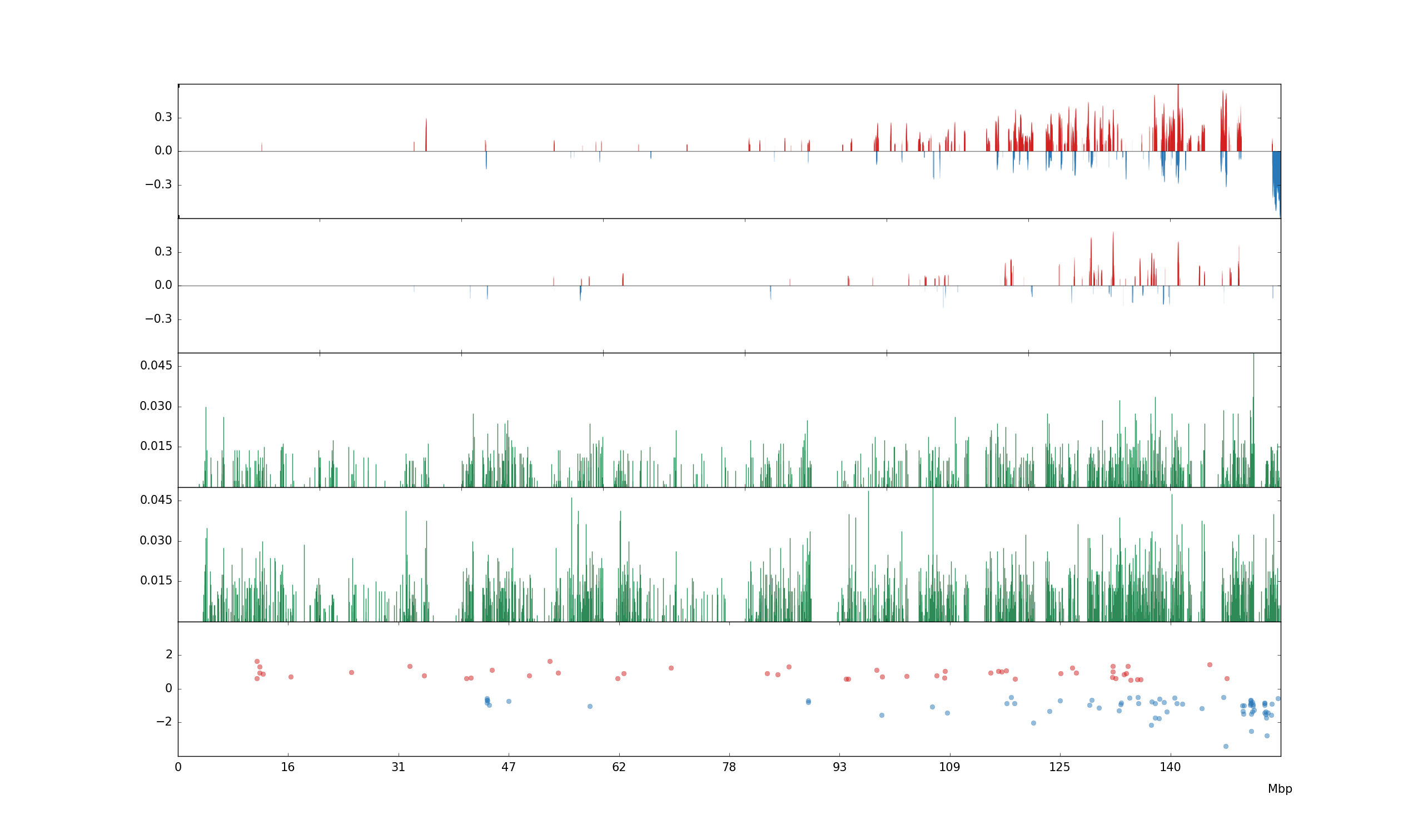


Figure S3.

## Positions of DIRs for all viewpoints 1, 2, 11 and 12 are compared against structural protein binding sites. First track is for *Df* and +*^129^* comparisons and second track is for $+_{D}^{Bl6}$ and +*^Bl6^* comparison. DIRs with increased/decreased contact probabilities compared to WT are plotted in red/blue. Shown in the third and fourth tracks (green bars) are the running average over 20Kb window for CTCF and Smc1 binding site footprint, respectively. The fifth track displays fold change for *Df/*$+_{D}^{Bl6}$ DE genes, color coded (red, over-expressed in *Df/*$+_{D}^{Bl6}$ MEFs; blue, over-expressed in +*^129^*/+*^Bl6^* MEFs).

## **Figure S4**

*
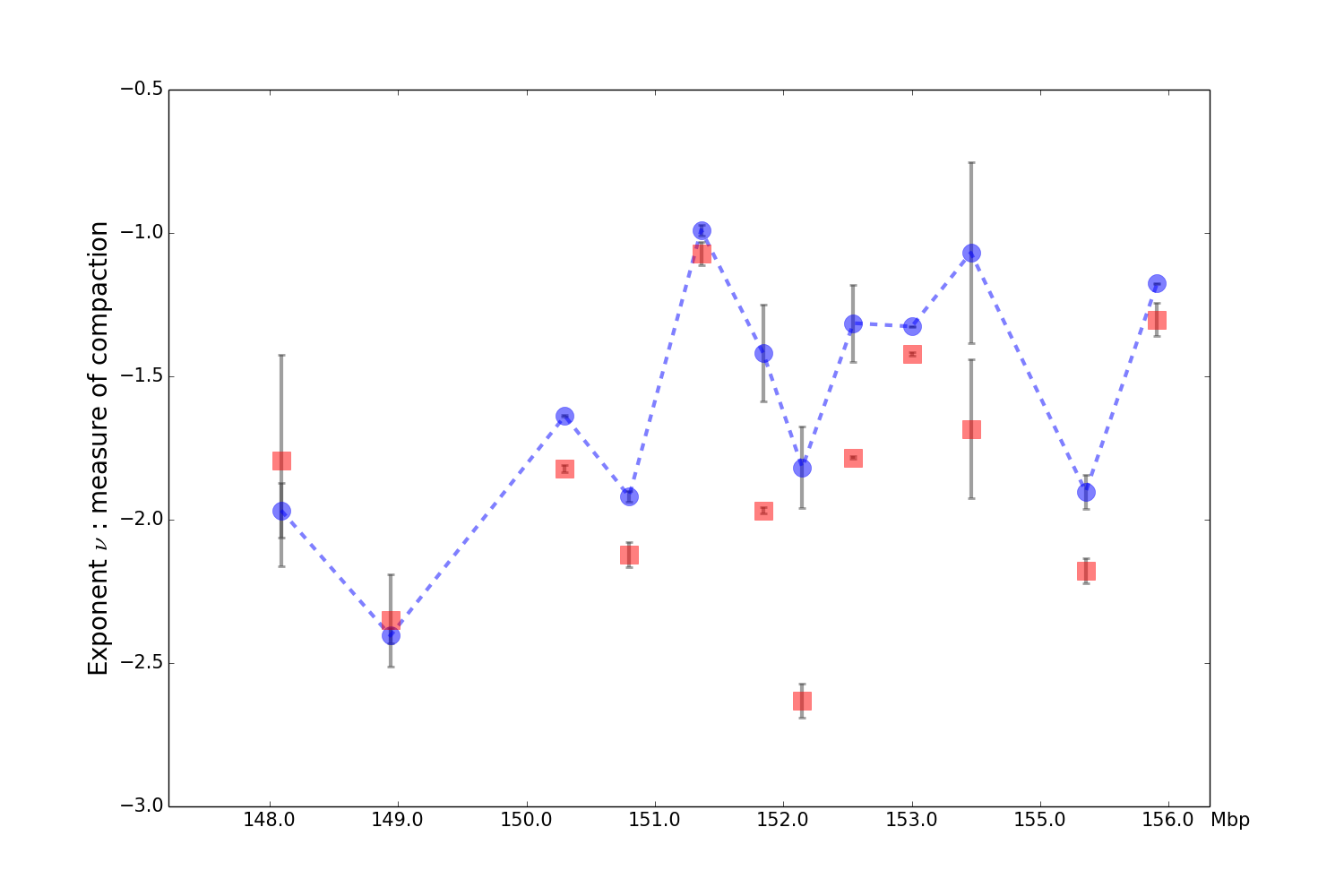
*

Figure S4. Calculated $\boldsymbol{\nu}$ per viewpoint for $\boldsymbol{+}_{\boldsymbol{D}}^{\boldsymbol{Bl}\boldsymbol{6}}$ vs +*^Bl6^*

$+_{D}^{Bl6}$ (red squares). +*^Bl6^* (blue circles). Error bars determined from the two available biological replicates. Notice there are no major differences between compaction values for both chromosomes, except for viewpoints 152.1 (viewpoint 6), 152.4 (viewpoint 7), and 152.9 (viewpoint 6), and 154.9 (viewpoint 11).

## **Figure S5**

**(A)** BACset1

*Df*


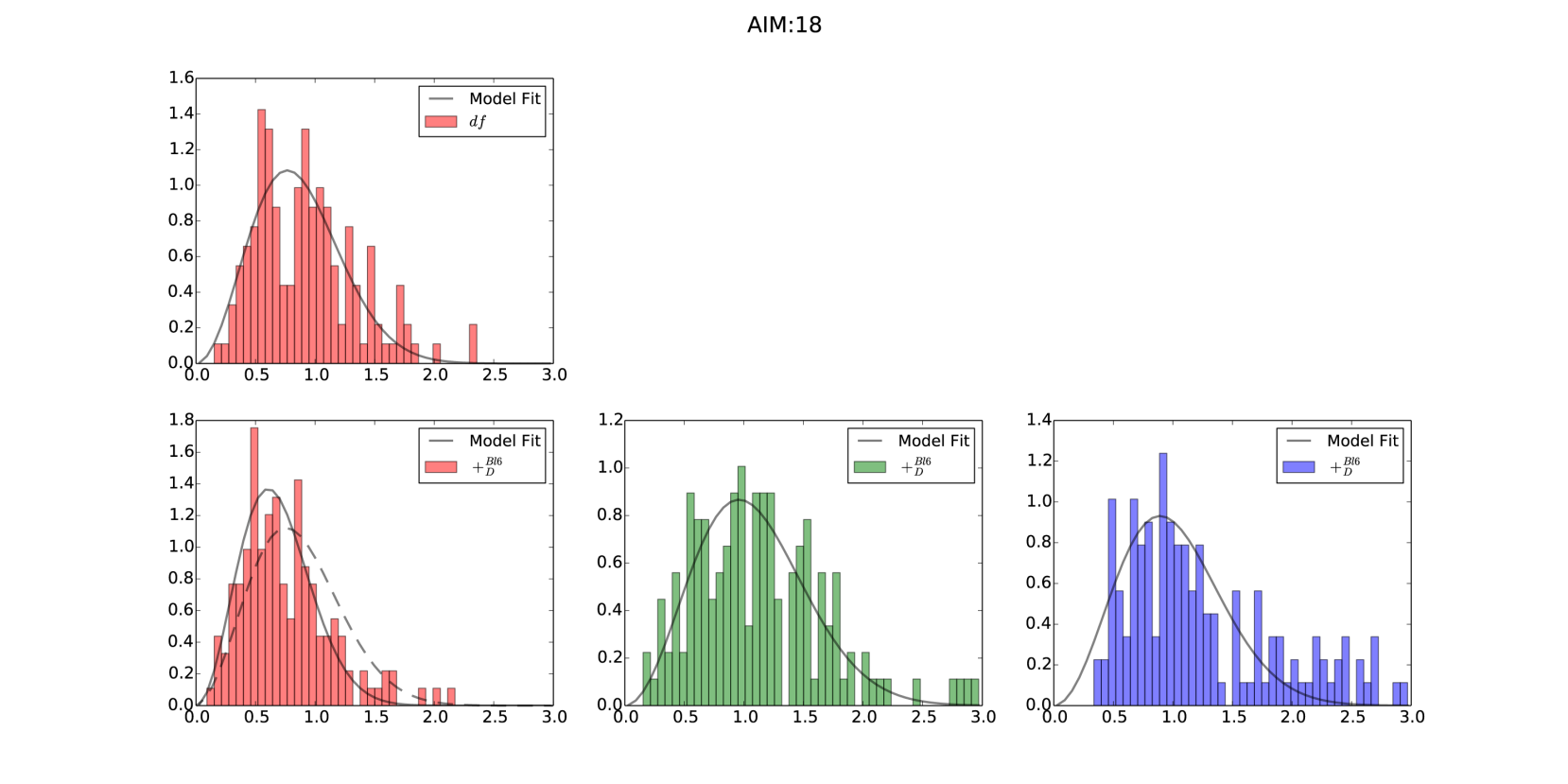


Combined WT


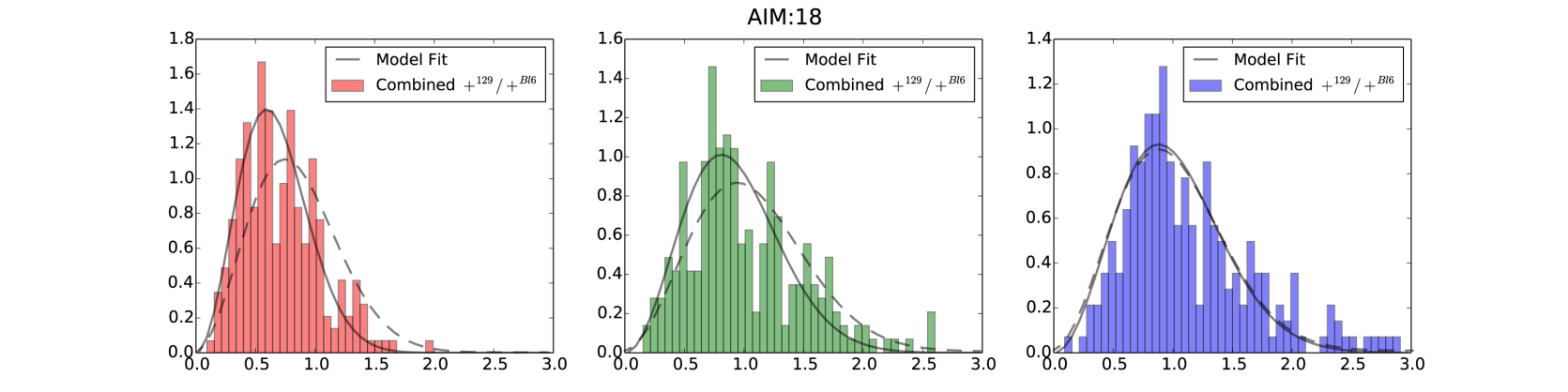


**(B)** BACset2


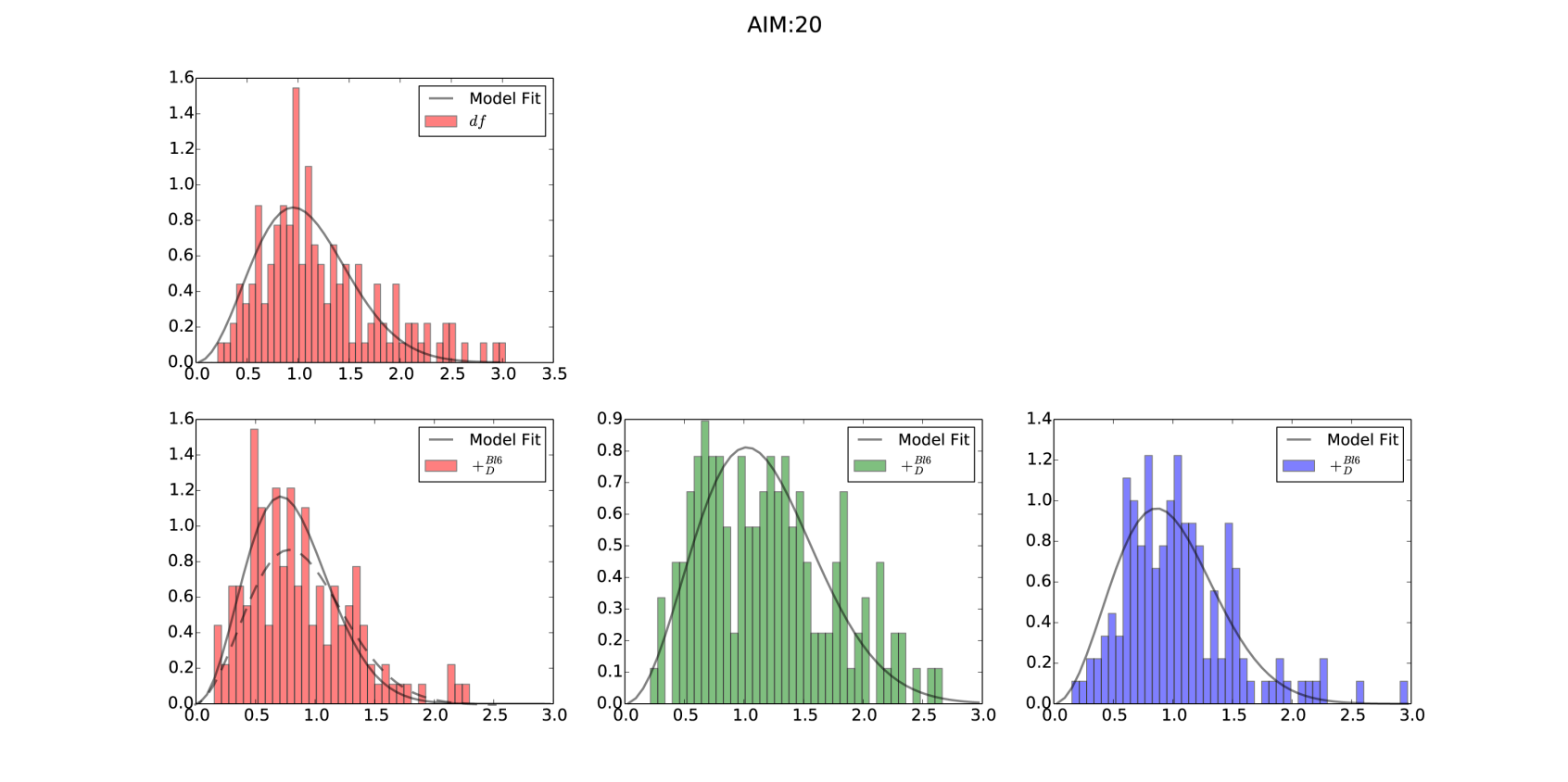
*Df*

Combined WT


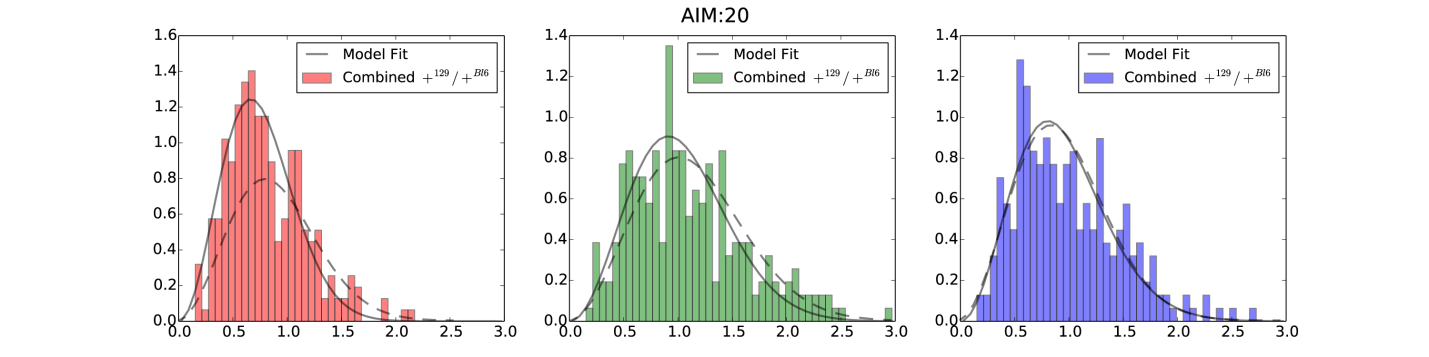


**(C)** BACset3

*Df*


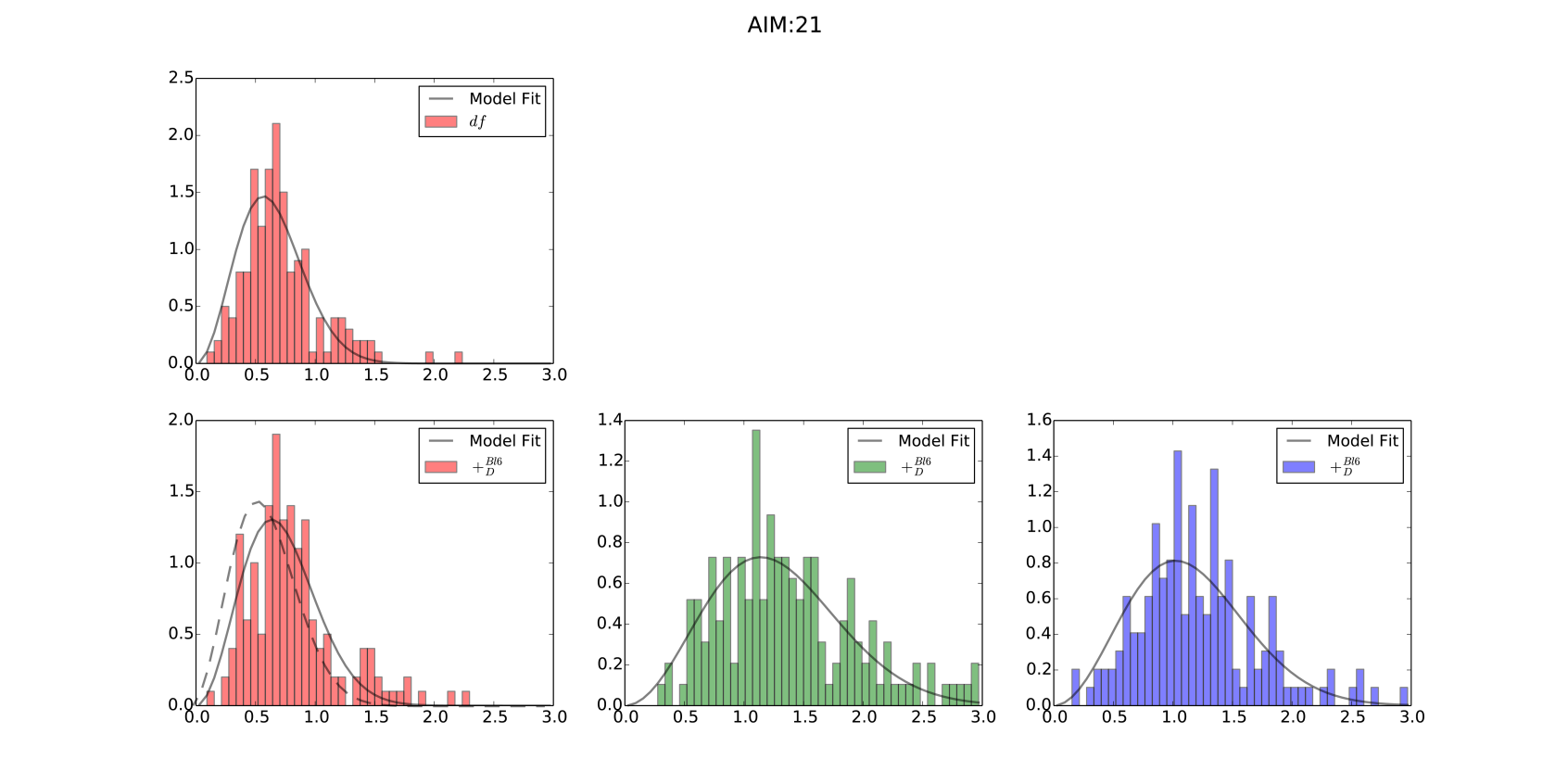


Combined WT


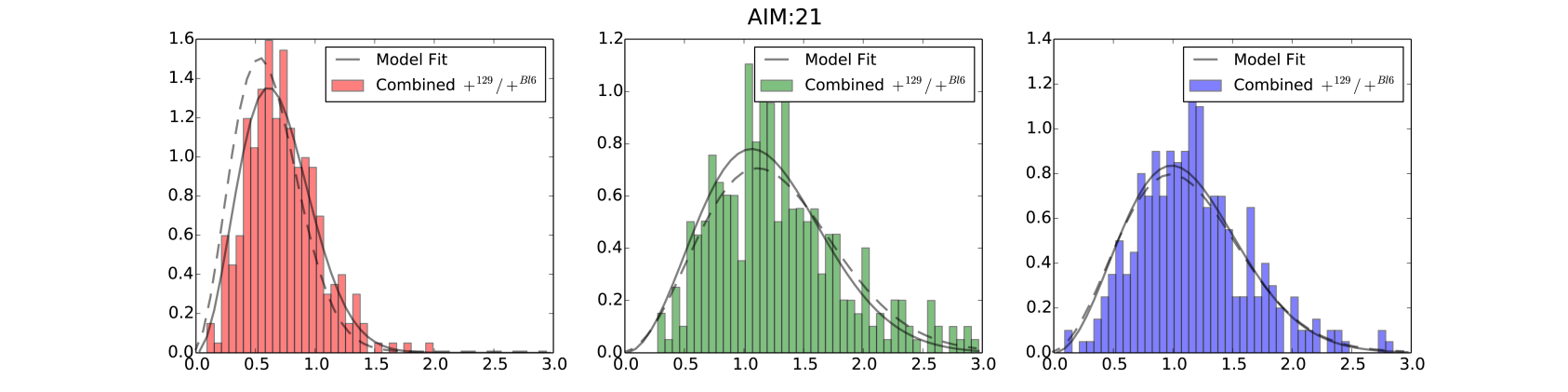


**(D)** BACset4

*Df*


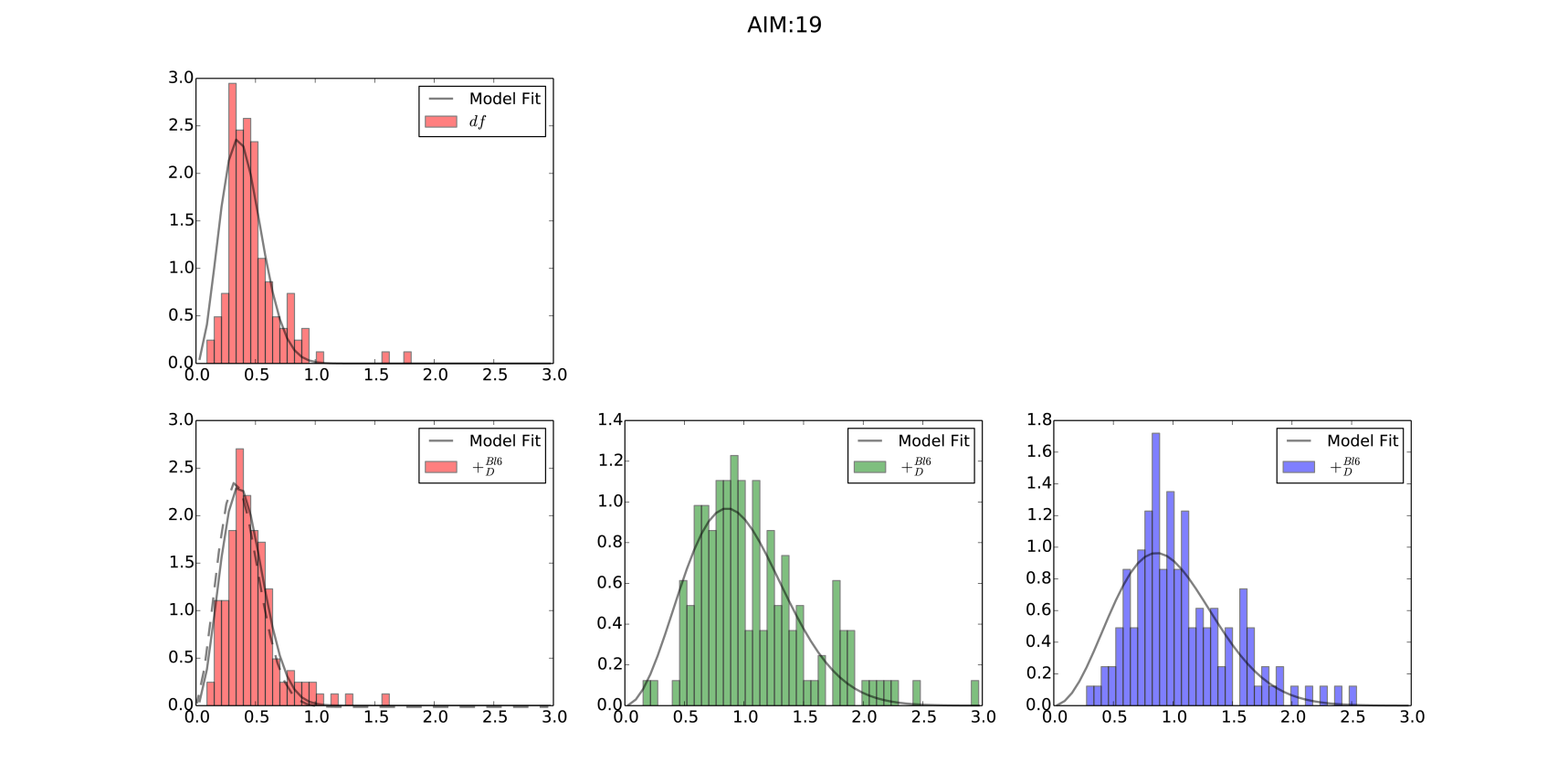


Combined WT


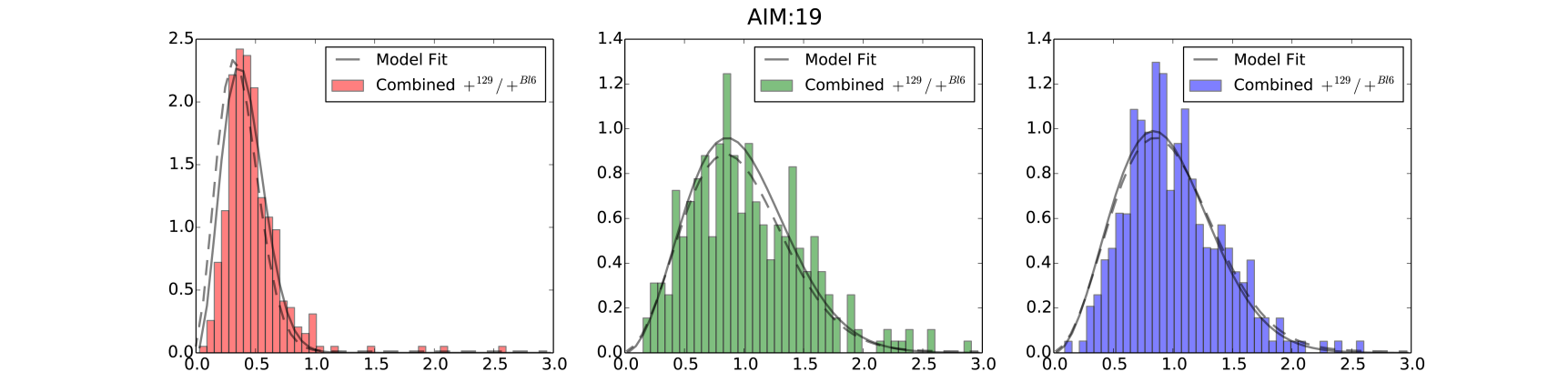


**Figure S5.** Distance distributions between query probes for (A) BACset1, (B) BACset2, (C) BACset3, (D) BACset4, FISH experiments. For each experiment, the two subfigures show the $Df/+_{D}^{Bl6}$ and $+^{129}/+^{Bl6}$ results. The $Df/+_{D}^{Bl6}$ sets: upper panel shows the distance distribution (upper panel solo) and fit for two of the query probes (1 and 2) in $Df.$Lower panel shows the distance distribution for all probe pairs in $+_{D}^{Bl6};$query probe 1 and query probe 2 (lower red), query probe 1 and deletion probe (green) and query probe 2 and deletion probe (blue). For the $+^{129}/+^{Bl6}$ set: we cannot distinguish between the two homologous chromosomes, so the average over them is shown; query probe 1 and query probe 2 (lower red), query probe 1 and deletion probe (green) and query probe 2 and deletion probe (blue). The differential analysis for $Df$is done using the fit to $Df$and the fit to the combined WT ($+^{129}/+^{Bl6}$). In the red histograms, the dashed line corresponds to the $Df$ model fit; green and blue histograms have a dashed model fit for $+_{D}^{Bl6}$probe distances.

# **Supplementary Tables**

Table S1. (a) Positions and (b) C57Bl6/J-129S5/SvEvBrd SNP information for analyzed PE-4Cseq viewpoints on chromosome 4. Primers used for Sanger sequencing are shown.

Table S2. Summary of median magnitude of change, direction, and number of *df* DIRs for viewpoints 1, 2, 11, and 12. Notice how viewpoint 1 displays an increase in contact probabilities with surrounding sequences (64% of total detected regions), while viewpoints 2 and 11 show mostly a decrease in interactions (60% for viewpoint 2, and 95% for viewpoint 11). Viewpoint 12 has both increase and decrease in interactions in approximately the same magnitude (~50%).

Table S3. Summary of median magnitude of change, direction, and number of $\boldsymbol{+}_{\boldsymbol{D}}^{\boldsymbol{Bl}\boldsymbol{6}}$ DIRs for viewpoints 1-12. Notice how only viewpoint 5 displays an increase in contact probabilities (63% of total regions). Viewpoints 3, 4, and 9 show a ~50% split between regions with an increase and decrease in contact probabilities, while the rest of the viewpoints show a variable range in the decrease in interactions (60-79% of total regions per viewpoint).

**Table S4. Unique (a) *df* and (b) *Df/***$\boldsymbol{+}_{\boldsymbol{D}}^{\boldsymbol{Bl}\boldsymbol{6}}$ **regions.** All chromosome 4 positions are shown.

Table S5. (a) Summary of *df* DIRs overlap with CTCF, Mediator, and cohesin binding sites. Column 1, *Region*, refers to the viewpoint assessed. Column 2, *diffsites*, refers to *df* DIRs. Column 4, *no. sites* and feature name corresponds to the number of DIRs that contain the specified genomic feature. Column 6, *bp sites* feature, presents the sum of DIRs bp which contain the specified feature. Column 8, *no.* features, indicates the number of features included inside DIRs. Percentages in columns 5, 7, and 9 are calculated based on the total number of regions or features in the preceding column. (b) Summary of *df* DIRs overlap with CTCF, Mediator, and cohesin binding sites. Column 1, *Region*, refers to the viewpoint assessed. Column 2, *diffsites*, refers to *df* DIRs. Column 4, *no. sites* and feature name corresponds to the number of DIRs that contain the specified genomic feature. Column 6, *bp sites* feature, presents the sum of DIRs bp which contain the specified feature. Column 8, *no.* features, indicates the number of features included inside the DIRs. Percentages in columns 5, 7, and 9 are calculated based on the total number of regions or features in the preceding column. (c) Summary of $\boldsymbol{+}_{\boldsymbol{D}}^{\boldsymbol{Bl}\boldsymbol{6}}$ DIRs overlap for viewpoints 1, 2, 11, and 12 with CTCF, Mediator, and Smc1 binding sites. Column identities are as described in (a). (d) Summary of Monte Carlo simulations for assessing statistical significance of protein binding overlaps for $\boldsymbol{+}_{\boldsymbol{D}}^{\boldsymbol{Bl}\boldsymbol{6}}$ DIRs for viewpoints 1, 2, 11, and 12. Column identities are as described in (b). Notice the significant p-values obtained for CTCF and Smc1 binding (p-val < 0.001, rounded down to zero in table).

Table S6. Sequencing LAD (sLAD) positions detected for the terminal part of chromosome 4. Data derived from 3T3 MEFs[^40^](#_ENREF_40). Marked in yellow is the extensive sequence stretch bordering the CNV devoid of sLADs. Marked in green are the sLAD regions inside the CNV.

Table S7. BACS used for selected PE-4Cseq and chromatin decompaction regions. a) Location of BACs used in 3D DNA FISH experiments for the validation of *df* PE-4Cseq DIRs and chromatin decompaction. b) Number of CTCF, Med1, Med12, and Smc1 protein binding sites[^33^](#_ENREF_33) overlapping each BAC used for 3D DNA FISH experiments. c) Proportions of CTCF, Med1, Med12, and Smc1[^33^](#_ENREF_33) protein binding sites overlapping each BAC used for 3D DNA FISH experiments normalized by BAC size.

Table S8. Combined DE genes of three +*^129^*/+*^Bl6^* and four *Df/*$\boldsymbol{+}_{\boldsymbol{D}}^{\boldsymbol{Bl}\boldsymbol{6}}$ MEF revealed by RNA-Seq.

Table S9. GO “condensed nuclear chromosome” table of DE genes in *df/+^Bl6^* MEFs

Table S10. 129S5/SvEvBrd allele-specific DE genes of three +*^129^*/+*^Bl6^* and four *Df/*$\boldsymbol{+}_{\boldsymbol{D}}^{\boldsymbol{Bl}\boldsymbol{6}}$ MEF revealed by RNA-Seq.

Table S11. C57Bl6/J allele-specific DE genes of three +*^129^*/+*^Bl6^* and four *Df/*$\boldsymbol{+}_{\boldsymbol{D}}^{\boldsymbol{Bl}\boldsymbol{6}}$ MEF revealed by RNA-Seq.

Table S12. Candidate genes associated with different Monosomy 1p36 phenotypes. Their corresponding mouse homologues are shown in column 3, together with their chromosomal positions (columns 5,6). Their overlaps with *df* DIRs are displayed in columns 7-9, as well as the direction of change of the contact probabilities (1= increase, -1=decrease. Both compared to +*^129^*). RNA-Seq derived expression in *Df/+^Bl6^* MEFs is shown in columns 11,12. Mouse gene coordinates are expressed in NCBI37/mm9 assembly, while human is GRCh38.

# **References**

1. Solovei, I. & Cremer, M. 3D-FISH on cultured cells combined with immunostaining. *Methods Mol Biol* **659**, 117-26 (2010).

2. Splinter, E. *et al.* The inactive X chromosome adopts a unique three-dimensional conformation that is dependent on Xist RNA. *Genes Dev* **25**, 1371-83 (2011).

3. Kagey, M.H. *et al.* Mediator and cohesin connect gene expression and chromatin architecture. *Nature* **467**, 430-5 (2010).

4. Quinlan, A.R. & Hall, I.M. BEDTools: a flexible suite of utilities for comparing genomic features. *Bioinformatics* **26**, 841-2 (2010).

5. Wu, F. & Yao, J. Spatial compartmentalization at the nuclear periphery characterized by genome-wide mapping. *BMC Genomics* **14**, 591 (2013).
